# Sitticine jumping spiders: phylogeny, classification, and chromosomes (Araneae, Salticidae, Sitticini)

**DOI:** 10.3897/zookeys.925.39691

**Published:** 2020-04-08

**Authors:** Wayne P. Maddison, David R. Maddison, Shahan Derkarabetian, Marshal Hedin

**Affiliations:** 1 Departments of Zoology and Botany and Beaty Biodiversity Museum, University of British Columbia, 6270 University Boulevard, Vancouver, British Columbia, V6T 1Z4, Canada University of British Columbia Vancouver Canada; 2 Department of Integrative Biology, Oregon State University, Corvallis, OR 97331, USA Oregon State University Corvallis United States of America; 3 Department of Biology, San Diego State University, San Diego, CA 92182, USA San Diego State University San Diego United States of America; 4 Department of Organismic and Evolutionary Biology, Harvard University, Cambridge MA 02138, USA Harvard University Cambridge United States of America

**Keywords:** Amycoida, karyotype, molecular phylogeny, Salticinae, sex chromosomes

## Abstract

The systematics of sitticine jumping spiders is reviewed, with a focus on the Palearctic and Nearctic regions, in order to revise their generic classification, clarify the species of one region (Canada), and study their chromosomes. A genome-wide molecular phylogeny of 23 sitticine species, using more than 700 loci from the arachnid Ultra-Conserved Element (UCE) probeset, confirms the Neotropical origins of sitticines, whose basal divergence separates the **new subtribe**Aillutticina (a group of five Neotropical genera) from the subtribe Sitticina (five genera of Eurasia and the Americas). The phylogeny shows that most Eurasian sitticines form a relatively recent and rapid radiation, which we unite into the genus *Attulus* Simon, 1868, consisting of the subgenera *Sitticus* Simon, 1901 (seven described species), *Attulus* (41 described species), and *Sittilong* Prószyński, 2017 (one species). Five species of *Attulus* occur natively in North America, presumably through dispersals back from the Eurasian radiation, but an additional three species were more recently introduced from Eurasia. *Attus palustris* Peckham & Peckham, 1883 is considered to be a full synonym of *Euophrys floricola* C. L. Koch, 1837 (not a distinct subspecies). *Attus sylvestris* Emerton, 1891 is removed from synonymy and recognized as a senior synonym of *Sitticus magnus* Chamberlin & Ivie, 1944. Thus, the five native *Attulus* in North America are *Attulus floricola*, *A. sylvestris*, *A. cutleri*, *A. striatus*, and *A. finschi*. The other sitticines of Canada and the U.S.A. are placed in separate genera, all of which arose from a Neotropical radiation including *Jollas* Simon, 1901 and *Tomis* F.O.Pickard-Cambridge, 1901: (1) *Attinella* Banks, 1905 (*A. dorsata*, *A. concolor*, *A. juniperi*), (2) *Tomis* (*T. welchi*), and (3) *Sittisax* Prószyński, 2017 (*S. ranieri*). All Neotropical and Caribbean “*Sitticus*” are transferred to either *Jollas* (12 species total) or *Tomis* (14 species). *Attinella* (three species) and *Tomis* are both removed from synonymy with *Sitticus*; the synonymy of *Sitticus cabellensis* Prószyński, 1971 with *Pseudattulus kratochvili* Caporiacco, 1947 is restored; *Pseudattulus* Caporiacco, 1947 is synonymized with *Tomis*. Six generic names are newly synonymized with *Attulus* and one with *Attinella*. Two Neotropical species are described as new, *Jollas cupreus***sp. nov.** and *Tomis manabita***sp. nov.** Forty-six new combinations are established and three are restored. Three species synonymies are restored, one is new, and two are rejected. Across this diversity of species is a striking diversification of chromosome complements, with X-autosome fusions occurring at least four times to produce neo-Y sex chromosome systems (X_1_X_2_Y and X_1_X_2_X_3_Y), some of which (*Sittisax ranieri* and *S. saxicola*) are sufficiently derived as to no longer preserve the simple traces of ancestral X material. The correlated distribution of neo-Y and a base autosome number of 28 suggests that neo-Y origins occurred preferentially in lineages with the presence of an extra pair of autosomes.

## Introduction

The jumping spider species long placed in the genus *Sitticus* Simon, 1901 are well known in both Eurasia and the Americas as prominent members of habitats as diverse as boreal forests, marshes, deserts and human habitations (e.g., Locket and Milledge 1951; Prószyński 1968, 1971, 1973, 1980; Harm 1973; Logunov and Marusik 2001). They belong to the tribe Sitticini, characterized morphologically by the loss of a retromarginal cheliceral tooth, long fourth legs, and an embolus fixed to the tegulum. Phylogenetic studies have suggested that sitticines arose in the Neotropics, dispersed to Eurasia, and radiated there (Maddison and Hedin 2003; Maddison 2015), a breadth of distribution rarely seen in recent lineages of salticids. The Neotropical sitticines (Figs 1–10) show considerable diversity, with some species having males with colourful and fringed courtship ornaments (*Aillutticus* Galiano, 1987; Figs 2, 3), and others with shiny metallic colours (*Jollas geniculatus* group; Figs 7, 113–116). The Eurasian radiation is more sedate in appearance, though there is still diversity in form and markings in *Attulus* Simon, 1868 (Figs 15–47).

**Figures 1–14.**
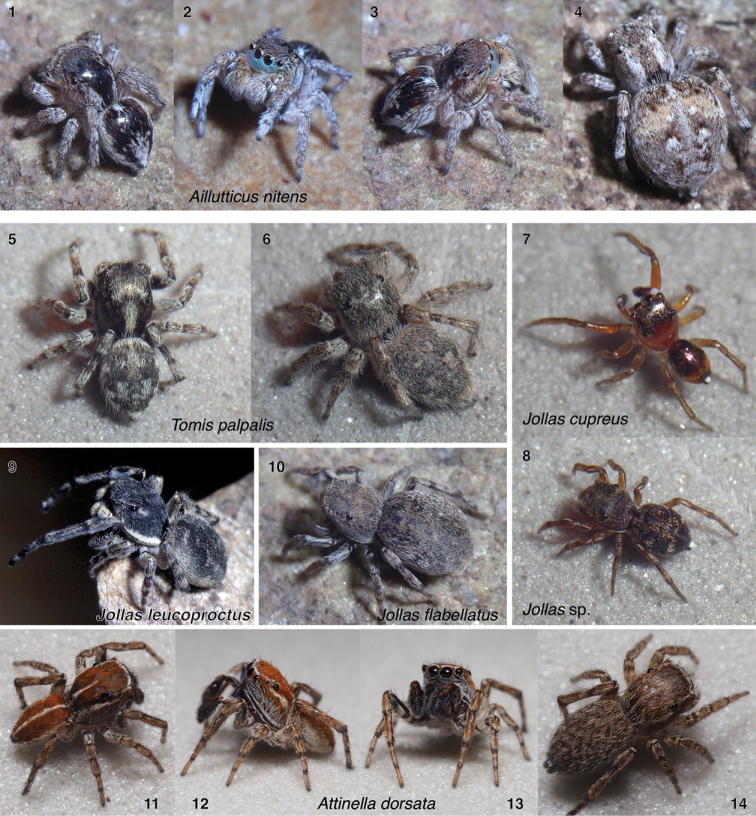
Subtribe Aillutticina (**1–4**) and the *Jollas*-*Tomis* clade of the subtribe Sitticina (**5–14**) **1–4***Aillutticus nitens*, Uruguay (-34.877, -56.023): **1–3** male **4** female **5, 6***Tomis palpalis* male and female, Ecuador (-0.1996, -77.7023) **7, 8***Jollas* species: **7***J. cupreus* male, Ecuador (-0.675, -76.397) **8***Jollas* sp. female, Ecuador (-0.7223, -77.6408) **9***J. leucoproctus*, Uruguay (-34.94, -54.95) **10***J. flabellatus*, Uruguay (-34.426, -55.195) **11–14***Attinella dorsata* male (**11–13**) and female (**14**), Canada (48.870, -123.379). Also included in the *Jollas*-*Tomis* clade is *Sittisax* (Figs 99–103). Additional members of the *Jollas*-*Tomis* clade can be seen in Figs 108–128.

This work’s three goals are to resolve sitticine phylogeny, to review the taxonomy of sitticines of one region (Canada), and to describe the remarkably diverse chromosomes of sitticines. Our immediate (and urgent) purpose in studying the group’s phylogeny is to settle its turbulent generic classification, which has seen, for instance, some well-known species change names three times in two years, for example, from *Sitticus floricola* (C. L. Koch, 1837) to *Sittiflor floricola* (by Prószyński 2017a) to *Calositticus floricola* (by Blick and Marusik 2018) and back to *Sitticus floricola* (by Breitling 2019).

Until the last few years, most sitticines were placed in the single widespread and species-rich genus *Sitticus* Simon, 1901 (e.g., Platnick 2014 listed 84 species). Prószyński, who developed our understanding of north-temperate species in a series of papers (1968, 1971, 1973, 1980), recently (2016, 2017a) partitioned this diversity into several genera: *Sittipub* Prószyński, 2016, *Sittiflor* Prószyński, 2017, *Sittilong* Prószyński, 2017, *Sittisax* Prószyński, 2017, *Sittiab* Prószyński, 2017, *Attulus*, and *Sitticus*. Prószyński did not intend this classification to be phylogenetic, but rather “pragmatic” (Prószyński 2017b), which is to say, not based on a conceptual justification. If a classification rejects reference to a broader theory, whether about monophyly or adaptive zones or predictivity across many characters, then it is not clear what it means, how it can be tested, or whether it can even be correct, except in its specific statements about the few characters mentioned. Furthermore, Prószyński provides little discussion of the diagnostic characters, indeed arguing against explicitly stating or explaining them (see Prószyński 2017a; Kropf et al. 2019). Thus, both his characters and his taxa remain inscrutable.

Breitling (2019) reversed Prószyński’s splitting by synonymizing many of the genera back into *Sitticus*, based on results from the single mitochondrial gene COI. We are fortunate that Breitling followed only a small fraction of the implications of his COI gene tree, for had they been followed more thoroughly they would have yielded taxonomic chaos in sitticines and throughout salticids, given that they scramble many well-supported salticid relationships, splitting (for instance) *Sitticus* sensu lato among five different tribes (discussed below, with our phylogenetic results). That COI is particularly bad at resolving salticid phylogeny has been reported previously (Hedin and Maddison 2001; Maddison et al. 2008, 2014; Bodner and Maddison 2012; Maddison and Szűts 2019). The results from this single mitochondrial gene therefore have given us no secure basis for sitticine taxonomy.

Neither Prószyński’s “pragmatic” classification nor Breitling’s COI-based classification have promoted taxonomic stability in sitticines. Prószyński’s intentionally non-phylogenetic approach is particularly problematical. The great majority of systematists no longer use such “pragmatic” non-evolutionary classifications, as they are not anchored to a broadly predictive external reality: they are subject to the whims of biologists’ interests and the character systems they focus on. A taxon delimited for this sense of pragmatism carries with it no promise of meaning or utility, other than the promise it will bear the diagnostic characters chosen. Different choices of diagnostic characters would lead to different classifications, with no basis for selecting among different authors’ approaches except the weight of authority – in the end, not as pragmatic as a stable phylogenetic classification, which, by the implications of genetic descent, will predict trait distributions across the genome. Breitling’s approach might have dampened the instability, as it is phylogenetic and uses explicit data and analysis, but his choice of the single gene COI, without supporting morphological information, has yielded a classification in which we can have little confidence. Prószyński’s and Breitling’s reclassifications might have been steps forward had they been done in a group of salticids with almost no previous attention, but the sitticines are reasonably well studied and often mentioned in the literature. These sudden, comprehensive, conflicting, and largely baseless rearrangements of *Sitticus* have yielded taxonomic instability in a well-known group.

Taxonomic instability yields confusion in ecological and other biodiversity literature about the identity of species studied, and damages the reputation of the taxonomic enterprise. We are now sufficiently capable of resolving phylogeny that we do not need to rely on the “pragmatic” choices of one authority or on a single misbehaving gene. Our goal is to provide stronger evidence, explicitly analyzed, for phylogenetic relationships in order to stabilize the classification of sitticines.

## Materials and methods

### Morphology

Preserved specimens were examined under both dissecting microscopes and a compound microscope with reflected light. Most of the coquille drawings were done in 1977 or 1978 using a reticle grid in a stereomicroscope. Colour drawings were done in 1974 through 1977 with a stereomicroscope and reticle grid. Pen and pencil drawings were made recently using a drawing tube on a Nikon ME600L compound microscope. Because some images were made decades ago, we are unable to supply scale bars on many. Terms used are standard for Araneae. All measurements are given in millimeters. Carapace length was measured from the base of the anterior median eyes not including the lenses to the rear margin of the carapace medially; abdomen length to the end of the anal tubercle. The following abbreviations are used: ALE, anterior lateral eyes; PLE, posterior lateral eyes; PME, posterior median eyes (the “small eyes”); RTA, retrolateral tibial apophysis of the male palp.

Specimens were examined from the collections of the American Museum of Natural History (**AMNH**), the Canadian National Collection of Insects, Arachnids and Nematodes (**CNC**), the Museo Argentino de Ciencias Naturales (**MACN**), the Museum of Comparative Zoology (**MCZ**), the Museum of Zoology, Pontificia Universidad Católica, Quito, Ecuador (**QCAZ**), and the Spencer Entomological Collection of the Beaty Biodiversity Museum (**UBC-SEM**).

### Nomenclatural authorities

Authors of nomenclatural acts in this paper vary by rank. For acts affecting the synonymy of genera (viz., reinstatement of *Attinella* and *Tomis*; synonymies of *Sitticus*, *Pseudattulus* and *Sittiab*), the authors are those of the paper itself. For all other acts, the author is W. Maddison. These include the establishment of the Aillutticina, new subtribe, acts that affect the synonymy and placement of species (new synonyms, restored synonyms, new combinations), and new species.

If not otherwise indicated, the authors of species names are given in the Classification section.

### Molecular phylogeny

Taxa were sampled to cover a diversity of sitticine species groups from Eurasia, North America, and South America (Table 1). Most were preserved in 95% ethanol, although we attempted to obtain sequences from some species (*Attulus rupicola*, *A. striatus*, *A. cutleri*) available only as 70–80% ethanol preserved specimens. We were unable to obtain sequences from *A. striatus* and *A. cutleri*, leaving us with a total of 23 sitticine species and two outgroups. The outgroups are *Breda*, from the sister group to sitticines, and *Colonus*, from the sister group to remaining amycoids as a whole (see Ruiz and Maddison 2015; Maddison et al. 2017).

**Table 1.** Specimens from which UCE sequence data gathered. “UCE loci” indicates number of loci from Phyluce. “Reads Pass QC” indicates number of reads retained after quality control and adapter removal via Illumiprocessor.

Species	Specimen	sex	Locality	Reads Pass QC	Contigs	UCE loci
*Aillutticus nitens*	d475	f	Uruguay: Canelones: -34.867, -56.009	946351	207743	434
*Attinella dorsata*	d490	m	U.S.A.: California: 37.2834, -120.8515	1617332	360661	480
*Attulus ammophilus*	d482	m	Canada: British Columbia: 49.7963, -119.5338	1471891	351670	588
*A. burjaticus*	RU18-7302	f	Russia: Tuva: 50.205, 95.135	529905	151897	627
*A. distinguendus*	RU18-6432	f	Russia: Tuva: 50.746, 93.142	406186	90846	626
*A. fasciger*	d487	m	Canada: Ontario: 43.35074, -79.75928	1370738	299273	564
*A. finschi*	d480	m	Canada: British Columbia: 49.0261, -114.0611	1489551	303924	579
*A. floricola*	d488	m	Canada: Saskatchewan: 52.4898, -107.3843	1466702	303612	606
*A. inexpectus*	RU18-6799	m	Russia: Tuva: 50.669, 92.9844	261947	60612	653
*A. longipes*	ARV4504	m	Italy: Stilfs	16385503	42677	515
*A. mirandus*	RU18-7308	f	Russia: Tuva: 50.205, 95.135	468358	110900	649
*A. pubescens*	d483	m	Canada: British Columbia: 49.2, -123.2	1316697	279173	503
*A. rupicola*	d491	m	Poland: Cisna near Lesko	187507	58418	312
*A. rupicola*	d492	m	Poland: Bukowksa Kopa	418777	137114	397
*A. saltator*	d512	m	Germany: Saxony: 51.607, 12.711	416618	113416	591
*A. sylvestris*	d489	m	U.S.A.: California: 36.3646, -121.5544	1289981	278727	506
*A. terebratus*	RU18-5346	m	Russia: Novosibirsk Oblast: 53.73, 77.866	306744	72547	668
*A. zimmermanni*	d493	m	Poland: Grabarka 52.417, 23.005	338718	113167	408
*A. zimmermanni*	RU18-5156	m	Russia: Novosibirsk Oblast: 53.721, 77.726	435654	93640	627
*Breda bicruciata*	d471	f	Uruguay: Lavalleja: -34.426, -55.195	646088	248616	549
*Colonus hesperus*	d472	m	U.S.A.: Arizona: 34.5847, -112.5707	1015130	250378	448
*Jollas cellulanus*	d479	f	Argentina: Neuquén: -37.0679, -69.7566	981935	268639	497
*J. cupreus*	d473	m	Ecuador: Orellana: -0.526, -77.418	1419103	289905	469
*J. cupreus*	d474	m	Ecuador: Orellana: -0.526, -77.418	3513351	723782	607
*J. leucoproctus*	d478	f	Uruguay: Maldonado: -34.94, -54.95	121131	61298	109
*Sittisax ranieri*	d481	m	U.S.A.: Oregon: 44.0322, -121.6722	1529835	322636	536
*Tomis manabita*	d476	m	Ecuador: Manabí: -1.5497, -80.8104	2524270	710859	651
*T. palpalis*	d477	m	Ecuador: Napo: -0.1996, -77.7023	1211674	256367	582

For most samples, DNA was extracted from multiple legs using the Qiagen DNeasy Blood and Tissue Kit (Qiagen, Valencia, CA) following manufacturer’s protocol. Specimens d491 and d492 of *Attulus rupicola* and d493 of *A. zimmermanni* were extracted using standard phenol-chloroform methods. UCE library preparation followed methods previously used in arachnids (e.g., Starrett et al. 2017; Derkarabetian et al. 2018; Hedin et al. 2018). Target enrichment was performed using the MYbaits Arachnida 1.1K version 1 kit (Arbor Biosciences; Faircloth 2017) following the Target Enrichment of Illumina Libraries v. 1.5 protocol (http://ultraconserved.org/#protocols). Libraries were sequenced with an Illumina HiSeq 2500 (Brigham Young University DNA Sequencing Center) with 150 bp paired end reads. Raw demultiplexed reads were processed with Phyluce (Faircloth 2016), quality control and adapter removal was conducted with the Illumiprocessor wrapper (Faircloth 2013), and assemblies were created with Velvet (Zerbino et al. 2008) at default settings. The *Sittilong longipes* ARV4504 sample was sequenced on a NovaSeq 6000 at the Bauer Core Facility at Harvard University with 150 bp paired end reads, and was assembled with Trinity (Grabherr et al. 2011) with default settings. Contigs were matched to probes using minimum coverage and minimum identity values of 80. UCE loci were aligned with MAFFT (Katoh and Standley 2013) and trimmed with Gblocks (Castresana 2000; Talavera and Castresana 2007), using -- b1 0.5, --b2 0.5, --b3 10, --b4 4 settings in the Phyluce pipeline.

In the resulting set of loci, most taxa have over 100,000 base pairs of sequence data, but some are less thoroughly sequenced. The less thoroughly sequenced taxa are: *J. leucoproctus* d478 (13,943 bp), *Attulus rupicola* d491 (46,660 bp), *Attulus rupicola* d492 (65,500 bp), and *A. zimmermanni* d493 (68,285 bp). The last species is represented by an alternative well-sequenced specimen, the others by well-sequenced close relatives. Although we did analyses with the entire set of taxa (“All Taxa”), we were concerned that the weakly sequenced taxa would disrupt resolution. Therefore, we rely primarily on analyses (and bootstrap values) that exclude these and use only the remaining well-sequenced taxa (“Core Taxa”). The Core Taxa dataset also excludes the less thoroughly sequenced of the two specimens of *Jollas cupreus* (d473, 92,549 bp).

This pipeline therefore resulted in two collections of genes, one of 968 loci for all the taxa (“All Taxa”), the other of 957 loci for the core set of well-sequenced taxa (“Core Taxa”). A filter of occupancy was then applied, eliminating all loci which had sequences for fewer than seven of the 20 well-sequenced taxa of the ingroup (*Jollas*, *Attinella*, *Tomis*, *Sittisax*, *Attulus*), resulting in 810 loci in the All Taxa dataset and 803 in the Core Taxa dataset. Preliminary analyses of these loci revealed some whose gene trees strongly suggested two paralogs or chimeras were included: a single very long branch isolating a few taxa (which for all other considerations and subsequent analyses showed no indication of being so distinctive or related to one another), whose sequences differed from the others extensively and consistently. Out of caution we chose to discard a locus if its preliminary gene tree (RAxML 8.2.8, Stamatakis 2014, single search, default settings) had the longest branch at least five times longer than the second longest branch. Inspection of the results indicated this matched approximately our subjective judgment of a strong suspicion of paralogy. This filter left 749 loci in the All Taxa dataset and 757 loci in the Core Taxa dataset.

Maximum likelihood phylogenetic analyses were run using IQ-TREE version 1.6.7.1 (Nguyen et al. 2015), run via the Zephyr package (version 2.11, Maddison and Maddison 2018a) of Mesquite (Maddison and Maddison 2018b). The data were analyzed both without partitions (“unpartitioned”) and partitioned by locus, allowing the possibility of separate rates and substitution models (Kalyaanamoorthy et al. 2017). We ran 50 separate search replicates for the maximum likelihood tree for the concatenated analysis. We performed a standard bootstrap analysis with 1000 replicates and the same model and partition settings.

A separate small phylogenetic analysis was done to explore the distinction in *Attulus floricola* between hemispheres, using data of other specimens in Genbank and BOLD (boldsystems.org), to blend with our data. Insofar as only COI barcode data are available online, and this gene struggles to reconstruct salticid phylogeny (Hedin and Maddison 2001; Maddison et al. 2008, 2014; Bodner and Maddison 2012; Breitling 2019; Maddison and Szűts 2019), we provided a skeletal constraint tree of our UCE specimens from which we could obtain COI data, so that the gene’s burden would be only to place the extra COI-only *floricola* group specimens on this skeleton. We obtained COI data for our UCE taxa by mining the UCE reads for COI-alignable bycatch. A local database was assembled in Geneious v11.0.4 comprising labeled Velvet UCE contigs for all sequenced taxa, then published *A. striatus* sequences (voucher BIOUG14302-A06) were used to query this local database using BLASTN (max e value of 1×10^-10^). Retaining only high-coverage sequences, we recovered COI bycatch for all taxa except for *A. saltator*, *A. inexpectus*, and *A. rupicola*. For *A. saltator* and *A. rupicola* we substituted COI data from Genbank from another geographically proximate specimen. The constraint tree was set to match Figure 48 in topology. Then, we added and aligned COI sequences of *A. floricola* from scattered locations, as well as specimens of *A. caricis* (from the Netherlands) and *A. sylvestris* (from Canada). (The latter were identified in BOLD as *A. rupicola*, but inspection of genitalic photographs courtesy of G. Blagoev shows they are *A. sylvestris*.) The gene tree was recontructed by RAxML (Stamatakis 2014), with codon positions as separate partitions, and using Figure 48 as a skeletal (partial) constraint tree.

Sequence reads are deposited in the Sequence Read Archive (BioProject submission ID PRJNA605426, http://www.ncbi.nlm.nih.gov/bioproject/605426). Alignments and trees are deposited in the Dryad data repository (https://doi.org/10.5061/dryad.cjsxksn2q).

### Chromosomes

Chromosomes were studied in 17 taxa of Sitticina. The specific identity of the specimen labelled “*A. rupicola*/*floricola*” is ambiguous because the voucher specimen has not been located, and the first author is not confident he was able to distinguish the two species in the 1980s. Although its specific identity is not known, it can be confidently placed within the *floricola* group, and so can play a role in phylogenetic interpretation.

Meiotic chromosomes were observed in testes of adult and subadult males using Feulgen staining, following the methods of Maddison (1982), except that no colchicine was used. Most preparations of Nearctic material were done between 1980 and 1989, and scored for autosome number and form and sex chromosome system soon thereafter. In the years since, some of the slides have faded considerably, and even with phase contrast they can no longer be scored. For most species we were able to confirm the old scores through re-examination (in an Olympus BX51 phase contrast microscope), except as noted in Chromosome observations. Because of the long history of this study, our photographs are of varied ages and qualities. We recognize that chromosome scoring of some species has uncertainty, and that future studies should be directed to confirming or correcting our intepretations. Nonetheless, the broad patterns we describe are supported even taking the uncertainty into account.

Evidence for scoring chromosome complement of each species is described in Chromosome observations. Most chromosome scoring was done from meiotic nuclei in first metaphase or diakinesis showing chromosomes that are well separated, or, if overlapping, easily interpretable. Although well-spread mitotic nuclei would have added useful data, we judge meiotic chromosomes to be sufficient as they show distinctive features, e.g. when they are oriented by the centromere pulling toward the pole on the metaphase plate. Metacentrics show an obvious bend at the centromere where the second arm hangs loose like a dog’s ear (Fig. 130), while acrocentrics show an opposite bend more distally (at chiasmata), or no bend (if chiasmata are terminal), and a narrower neck to the centromere stretched pole-ward (Figs 131, 140, 143, 147, 154, 156, 164). In most specimens, multiple nuclei contributed to the scoring. In other salticids (e.g., Maddison 1982), the Xs of the X_1_X_2_0 sex chromosome system have distinctive behaviour during meiosis. At first metaphase they typically lie toward one pole, side by side and without chiasmata. They are heteropycnotic, condensing early, but by first metaphase slightly decondensed, and in second prophase condensed. We use this behaviour as evidence for interpreting chromosomes as Xs, or for interpreting portions of chromosomes as representing ancestral X material. For several species additional evidence came from metaphase II counts, and for one (*Sittisax ranieri*) female mitosis in subadult digestive glands was examined.

In describing chromosome complements, we use “a” and “m” to indicate one-armed (acrocentric/telocentric) and two-armed (metacentric/submetacentric) chromosomes respectively. Thus, “26a+XaXa0” would mean “26 acrocentric autosomes plus two X’s, both of which are acrocentric”. In all cases, the multiple Xs of a male are interpreted as not being homologous, and therefore it would be more proper to refer to the systems as X_1_X_2_0, X_1_X_2_Y, or X_1_X_2_X_3_Y rather than as XX0, XXY, or XXXY. However, the “1”, “2”, “3” will be left implicit, omitted for ease of reading, to avoid overly complex labels like Xa_1_Xa_2_Xa_3_Ym.

### Phylogenetic results

The maximum likelihood tree from the UCE data is shown in Figure 48, which incorporates results from both partitioned and unpartitioned analyses. As seen in previous results from fewer genes (Ruiz and Maddison 2015), *Aillutticus* Galiano, 1987 is the sister group to all other sitticines sampled. *Aillutticus* is the only sampled representative of what is likely a large radiation of little-studied Neotropical sitticines with high, rounded carapaces and unusual genitalia, currently including five genera (Galiano 1987; Ruiz and Brescovit 2005, 2006). As described under classification, we propose the name Aillutticina, new subtribe, for the *Aillutticus* group of genera, and the name Sitticina for the remaining sitticines.

The phylogeny of Sitticina shows two major groups, the *Jollas*-*Tomis* clade and *Attulus*. The *Jollas*-*Tomis* clade is distributed entirely in the Americas except for the two species of *Sittisax*; *Attulus* is entirely Eurasian except for 8 species in North America. The only previously published comprehensive phylogeny of sitticines, of Prószyński (1983), is substantially similar in placing *Sittisax* and *Attinella* outside of the major clade of the *floricola*, *distinguendus* and *penicillatus* species groups. The most notable differences between his arrangement and ours are the placements of *Attulus pubescens* and *A. dzieduszycki*. Prószyński’s more recent (2017a) classification into genera, however, is discordant in many respects with our results, as can be seen in the many combinations that we establish or reinstate below in order to achieve monophyly of genera and subgenera. This discord may have arisen partly because Prószyński was not attempting to create a taxonomy that reflected phylogenetic relationships, but rather the distribution of a few diagnostic characters (Prószyński 2017b).

Our UCE phylogeny differs in several respects from Breitling’s (2019) COI phylogeny. Ours places *Sittisax ranieri* next to *Tomis*, distant from the Eurasian Radiation, while his places it next to *Attulus finschi*. The other disagreements are not visible in the isolated portion of the tree shown in Breitling’s figure 9B, but are visible in his more complete supplemental figure “Salticidae”. It places *Attinella concolor* sister to the euophryine *Sidusa*, *Attulus fasciger* among the plexippines, *Tomis manabita* (“*Sitticus* sp. MCH−2003”) as sister to the asemoneine *Asemonea*, and *Jollas cupreus* as sister to the lapsiine *Thrandina* – thus mixing the sitticines among three different subfamilies and 5 tribes. Given our far stronger data (hundreds of loci, multiple linkage groups, many times more nucleotide sites), inclusion of more Neotropical sitticines, more efficient analysis (likelihood as opposed to neighbour joining), and concordance with morphological traits uniting the sitticines, we consider Breitling’s phylogeny to be in error. The startling scrambling of established clades in Breitling’s supplemental figures is in accord with previous studies in salticids, which have shown the COI gene to be particularly error-prone in reconstructing phylogeny (Hedin and Maddison 2001; Maddison et al. 2008, 2014; Bodner and Maddison 2012; Maddison and Szűts 2019). However, our phylogeny agrees with one important result from Breitling’s study: the close relationship of *A. pubescens* with *A. terebratus* (though, as noted above, their close relative *A. fasciger* is placed in another tribe).

Although *Attulus* includes some Nearctic members, it is considerably more species-rich in Eurasia, and is most parsimoniously interpreted as having radiated there. The few Nearctic members of this clade are likely recent returns from the Palearctic, insofar as they are Holarctic (*Attulus floricola*, *A. cutleri*, *A. finschi*), close relatives of Eurasian species (*A. sylvestris* within the *A. floricola* group, *A. striatus* close to *A. rivalis*), or recent introductions (*A. ammophilus*, *A. fasciger*, *A. pubescens*: see Prószyński 1976, 1983 and Cutler 1990). Our results thus support Prószyński’s (1983) hypothesis of a Palearctic radiation of *Sitticus* sensu lato, although we differ in concluding that only one subgroup diversified in Eurasia, *Attulus*, arising from an earlier Neotropical diversification.

The deep branches of the Eurasian Radiation are short, suggesting the group diversified rapidly. Nonetheless, the monophyly of subgenus Sitticus is well supported by a bootstrap percentage of 100 in our primary Core Taxa analysis (Fig. 48). The monophyly of subgenus Attulus has weak bootstrap support in the partitioned analysis (72%), although good support in the unpartitioned analysis (95%). As well, the major subgroup of subgenus Attulus excluding *A. saltator* and *A. mirandus* is well supported (92% or 96%). Despite its weak support in the partitioned analysis, monophyly of subgenus Attulus as a whole is consistent across multiple analyses, for example, when the filter for loci present in at least seven core taxa is changed from seven to four or ten. Analyses (following the same methods described above) without *A. longipes* gave 99.6% bootstrap support to subgenus Attulus.

The relationships among *Attulus* species are concordant with morphological expectations with one exception: the placement of *A. burjaticus* with *A. zimmermanni*, suggesting that the longer embolus of *A. zimmermanni* and the *floricola* group are convergent. Otherwise, the *floricola* group holds together, as do the morphologically similar pairs of *A. ammophilus/distinguendus* and *mirandus/saltator*. The placement of *A. pubescens* nested within the *terebratus* group indicates that the very short embolus of the former is a derivation from the very long embolus of the latter.

*Jollas* and *Tomis* together form a Neotropical radiation and share (typically) an RTA that appears displaced basally, so as to appear to arise closer to the patella, as well as anteriorly placed epigynal openings.

## Classification

The phylogenetic results lead us to revise the generic division of sitticines. Unless we are to put all Sitticina into a single genus, perhaps palatable for the shallow-diverging Eurasian fauna, but not for the deep Neotropical lineages, then *Tomis* must be restored for many of the Neotropical species. Given that, *Sittisax* must be separated from *Attulus*/*Sitticus*, rejecting Breitling’s synonymy of this taxon with *Sitticus*. These choices are relatively easy. The more difficult choices concern the Eurasian Radiation.

Here we give a taxonomic review of the tribe, focussing especially on the species in Canada, and the two new species used in the molecular phylogeny (*Jollas cupreus* and *Tomis manabita*). In order to faciliate the use of figures for identification and comparison of species in North America, the sequence of taxa in figures will be different from that in the text, with a series of standardized plates placing images of all of the Canadian species in a block (Figs 49–103).

### Tribe Sitticini Simon, 1901

Amycoid salticids with fourth legs much longer than third and retromarginal cheliceral tooth lacking. Ancestrally they were ground-dwellers in the Neotropics, later diversifying in Eurasia to include species that live on tree trunks (e.g., *A. finschi*) and up in vegetation (e.g., *Attulus floricola*).

Eleven genera are here recognized in the Sitticini, including one (*Semiopyla* Simon, 1901) whose placement is unclear, and thus remains *incertae sedis* within the tribe. Two genera are in Eurasia (*Attulus* and *Sittisax*), while a disjunct set of eight genera are in South America (the five aillutticines, plus *Tomis*, *Jollas*, and *Semiopyla*). This geographical partitioning matches a phylogenetic division approximately, but not precisely, for the Holarctic *Sittisax* is phylogenetically a member of the Neotropical radiation. North America has four genera, one arising from the Eurasian radiation (*Attulus*), and three from the Neotropical radiation (*Attinella*, *Sittisax*, and *Tomis*).

Despite the synonymy of *Sitticus* with *Attulus*, the names Sitticini and Sitticina can persist (ICZN Article 40.1).

#### Subtribe Aillutticina

Taxon classificationAnimaliaAraneaeSalticidae

W. Maddison new subtribe

8304C37F-6464-5FDD-92FF-B463E932BD11

http://zoobank.org/4DBE8F82-300A-4AE0-9A11-7A0DC55D7099

Figures 1–4

##### Type genus.

*Aillutticus* Galiano, 1987

##### Diagnosis.

This group of five Neotropical genera was first recognized by Ruiz and Brescovit (2005, 2006), who characterize it as sharing “a high, broad carapace, laterally rounded behind the posterior lateral eyes, and the slightly convex dorsal surface of the cephalic region”. The contained genera are:

*Aillutticus* Galiano, 1987

*Amatorculus* Ruiz & Brescovit, 2005

*Capeta* Ruiz & Brescovit, 2005

*Gavarilla* Ruiz & Brescovit, 2006

*Nosferattus* Ruiz & Brescovit, 2005

#### Subtribe Sitticina Simon, 1901

There are no known morphological synapomorphies of this subtribe, but the molecular data show clearly that the five genera listed here form a clade. There are two major subgroups according to the UCE phylogeny: the genus *Attulus*, a primarily Eurasian radiation, and the *Jollas*-*Tomis* clade (*Attinella*, *Jollas*, *Sittisax*, *Tomis*), a primarily Neotropical radiation. We divide the taxonomy below into those two major groups, and under each discuss the genera, describe the Canadian species and two new Ecuadorian species used in the molecular work.

##### Genus *Attulus* Simon, 1868, restored (to respect its priority over *Sitticus*)

*Attulus* Simon, 1868 (type species *Attus helveolus* Simon, 1871)

*Sitticus* Simon, 1901 (type species *Araneus terebratus* Clerck, 1757)

*Sitticulus* F. Dahl, 1926 (type species *Attus saltator* O. Pickard-Cambridge, 1868), syn. nov.

*Calositticus* Lohmander, 1944 (type species *Attus caricis* Westring, 1861), syn. nov.

*Hypositticus* Lohmander, 1944 (type species *Aranea pubescens* Fabricius, 1775), syn. nov.

*Sittipub* Prószyński, 2016 (type species *Aranea pubescens* Fabricius, 1775), syn. nov.

*Sittiflor* Prószyński, 2017 (type species *Euophrys floricola* C.L. Koch, 1837), syn. nov.

*Sittilong* Prószyński, 2017 (type species *Attus longipes* Canestrini, 1873), syn. nov.

We unite the primary Eurasian radiation under the single genus *Attulus* because of the recency of the radiation, the very short phylogenetic branches separating the subgroups, and the clade’s morphological homogeneity. The total phylogenetic depth of *Attulus* is far less than that of its sister group (Fig. 48), but more importantly, the deepest branches of *Attulus* are very short. This suggests a rapid radiation, and that any subgroups will have only limited predictive value about traits, as most of the divergence occurred since the initial radiation. The monophyly of the major subgroups is to some extent uncertain, and so any generic division could be unstable. The morphological diversity encompassed by *Attulus* (e.g. variation in narrowness of carapace, leg length, embolus length, position of epigynal openings) is arguably less than that of other stable genera like *Pellenes* and *Habronattus*; the subgenera we recognize are comparable to species groups in *Habronattus* (Maddison and Hedin 2003) or subgenera in *Pellenes* (Logunov et al. 1999). By considering *Attulus* as a single genus with subgenera, we also simplify identifications by ecologists and others. A Eurasian salticid, even a juvenile, can easily be keyed to *Attulus* based on the long fourth legs and absence of retromarginal cheliceral teeth, except only for the exclusion of *Sittisax*.

Our choice to consider all but two Eurasian species as belonging to *Attulus* is informed partly by their phylogenetic context among Neotropical salticids. From a Palearctic perspective, the Eurasian radiation of sitticines may seem to represent a lineage of salticids so distinctive and species-rich that they deserve splitting into many genera, especially since the sister group of sitticines among the Old World salticids is the huge clade Salticoida (Maddison 2015), which is divided into hundreds of genera. From the Americas, though, the Eurasian sitticine radiation appears as a shallow expatriate lineage, the tip of the iceberg of a large and deeply diverging Neotropical radiation (the Sitticini, and more broadly, the Amycoida). If more generic subdivision is needed, it will be in the much more divergent and poorly explored sitticine fauna of South America.

The appropriate name for this unified genus is *Attulus*, as it is far older than *Sitticus*, and has been used continuously, though for only a few species. Two proposals have been made to ignore priority and instead use *Sitticus*, the generic name used for most of the species until Prószyński’s (2016, 2017) splitting. Prószyński himself had proposed to the ICZN in 2008 suppression of *Attulus* in favour of *Sitticus*, but in 2018 apparently withdrew that proposal (ICZN 2018). Breitling (2019) also proposed that the younger name *Sitticus* be used. We argue that priority in general should be respected unless it would disrupt a long-stable name against a little-used alternative. In this case, *Sitticus* has already been destabilized, *Attulus* has been used more or less continuously, and most species have already been moved to *Attulus* by Prószyński. The World Spider Catalog (WSC 2019) and other resources (Metzner 2019) have already begun to use *Attulus* for most species. Abandoning nomenclatural rules to avoid facing the consequences of new information will over the long term likely lead to instability or to classifications based on the weight of authority, just as with abandoning monophyly. Thus, the least disruptive choice is to use the name “*Attulus*”.

However, there is value in offering a weaker recognition of three subgroups of *Attulus*, as subgenera, given that there are names available: *Attulus*, *Sitticus*, and *Sittilong*. Our results support reciprocal monophyly of the subgenera *Attulus* and *Sitticus*, and a placement of Sittilong outside of both. Monophyly of subgenus Attulus has variable bootstrap support (72% to 95%, Fig. 48), although the clade’s presence is consistent across various alternative analyses (when *Sittilong* is not included; when the filter for loci present in at least seven core taxa is changed to four or ten). Even if subgenus Attulus falls apart with more data, the bulk of the subgenus would likely hold together, as there is high bootstrap support for the large subclade including the type species *A. distinguendus*. The low bootstrap support for the subgenus as a whole (in the partitioned analysis) derives from the weakness of inclusion of the unusual *penicillatus* group (represented by *A. saltator* and *A. mirandus*; see Logunov 1993), which might eventually need a separate subgenus (for which a name, *Sitticulus* F. Dahl 1926, is available).

The three subgenera have subtle but mostly consistent morphological differences. *Attuluss. str.* tends to have smaller and more compact bodies, with roundish carapaces (Figs 15–38). *Sitticus* have a narrower carapace and longer legs (Figs 39–47), and (except in *A. relictarius*) a large sweeping retrolateral tibial apophysis (Figs 74, 79, 84). *Sittilong* is notable for its long first legs.

*Attulus* includes 49 species in three subgenera:

Subgenus Attulus Simon, 1868, with 41 species:

Attulus (Attulus) albolineatus (Kulczyński, 1895), comb. nov., transferred from *Sitticus*

Attulus (Attulus) ammophilus (Thorell, 1875)

Attulus (Attulus) ansobicus (Andreeva, 1976)

Attulus (Attulus) atricapillus (Simon, 1882), comb. nov., transferred from *Calositticus*

Attulus (Attulus) avocator (O. Pickard-Cambridge, 1885)

Attulus (Attulus) barsakelmes (Logunov & Rakov, 1998), comb. nov., transferred from *Sitticus*

Attulus (Attulus) burjaticus (Danilov & Logunov, 1994)

Attulus (Attulus) caricis (Westring, 1861), comb. nov., transferred from *Calositticus*

Attulus (Attulus) clavator (Schenkel, 1936)

Attulus (Attulus) cutleri (Prószyński, 1980), comb. nov., transferred from *Calositticus*

Attulus (Attulus) damini (Chyzer, 1891)

Attulus (Attulus) distinguendus (Simon, 1868) (= type species *Attus helveolus* Simon, 1871)

Attulus (Attulus) dubatolovi (Logunov & Rakov, 1998)

Attulus (Attulus) dudkoi (Logunov, 1998), comb. nov., transferred from *Calositticus*

Attulus (Attulus) dzieduszyckii (L. Koch, 1870), comb. nov., transferred from *Sittisax*

Attulus (Attulus) eskovi (Logunov & Wesolowska, 1995), comb. nov., transferred from *Sitticus*

Attulus (Attulus) floricola (C. L. Koch, 1837), comb. nov., transferred from *Calositticus*

Attulus (Attulus) goricus (Ovtsharenko, 1978)

Attulus (Attulus) hirokii Ono & Ogata, 2018

Attulus (Attulus) inexpectus (Logunov & Kronestedt, 1997), comb. nov., transferred from *Calositticus*

Attulus (Attulus) inopinabilis (Logunov, 1992)

Attulus (Attulus) karakumensis (Logunov, 1992)

Attulus (Attulus) kazakhstanicus (Logunov, 1992)

Attulus (Attulus) mirandus (Logunov, 1993)

Attulus (Attulus) monstrabilis (Logunov, 1992), comb. nov., transferred from *Calositticus*

Attulus (Attulus) nenilini (Logunov & Wesolowska, 1993)

Attulus (Attulus) nitidus Hu, 2001, comb. nov., transferred from *Sitticus*

Attulus (Attulus) niveosignatus (Simon, 1880)

Attulus (Attulus) penicillatus (Simon, 1875)

Attulus (Attulus) penicilloides (Wesolowska, 1981)

Attulus (Attulus) pulchellus (Logunov, 1992), comb. nov., transferred from *Calositticus*

Attulus (Attulus) rivalis (Simon, 1937), comb. nov., and removed from synonymy with *A. striatus* (Emerton).

Attulus (Attulus) rupicola (C. L. Koch, 1837), comb. nov., transferred from *Calositticus*

Attulus (Attulus) saltator (O. Pickard-Cambridge, 1868)

Attulus (Attulus) sinensis (Schenkel, 1963)

Attulus (Attulus) striatus (Emerton, 1911), comb. nov., transferred from *Calositticus*

Attulus (Attulus) sylvestris (Emerton, 1891), comb. nov., transferred from *Sitticus*, removed from synonymy with *A. palustris*

Attulus (Attulus) talgarensis (Logunov & Wesolowska, 1993)

Attulus (Attulus) vilis (Kulczyński, 1895)

Attulus (Attulus) zaisanicus (Logunov, 1998)

Attulus (Attulus) zimmermanni (Simon, 1877), comb. nov., transferred from *Calositticus*

Subgenus Sitticus Simon, 1901, with seven species:

Attulus (Sitticus) fasciger (Simon, 1880), comb. nov., transferred from *Sitticus*

Attulus (Sitticus) finschi (L. Koch, 1879), comb. nov., transferred from *Sitticus*

Attulus (Sitticus) godlewskii (Kulczyński, 1895), comb. nov., transferred from *Sitticus*

Attulus (Sitticus) pubescens (Fabricius, 1775), comb. nov., transferred from *Sitticus*

Attulus (Sitticus) relictarius (Logunov, 1998), comb. nov., transferred from *Sitticus*

Attulus (Sitticus) tannuolana (Logunov, 1991), comb. nov., transferred from *Sitticus*

Attulus (Sitticus) terebratus (Clerck, 1757) (type species of *Sitticus*), comb. nov., transferred from *Sitticus*

Subgenus Sittilong Prószyński, 2017, with one species:

Attulus (Sittilong) longipes (Canestrini, 1873) (type species of *Sittilong*), comb. nov., transferred from *Sittilong*

###### Subgenus Attulus Simon, 1868

Figures 15–38, 49–73

*Attulus* Simon, 1868 (type species *Attus helveolus* Simon, 1871).

*Sitticulus* F. Dahl, 1926 (type species *Attus saltator* O. Pickard-Cambridge, 1868).

*Calositticus* Lohmander, 1944 (type species *Attus caricis* Westring, 1861).

*Sittiflor* Prószyński, 2017 (type species *Euophrys floricola* C.L. Koch, 1837).

Body generally more compact than in subgenus Sitticus, with a wider carapace. The spermatheca is a simple tube, folded near the middle. From the point at which the copulatory ducts enter the spermatheca, the spermatheca extends medially to the fertilization duct, but also laterally and then posteriorly (*floricola* group) or medially (most others) to a separate posterior lobe. Most Attulus (Attulus) have the embolus short, arising near the basal prolateral corner of the bulb, and the tegulum with basal edge more or less straight (not rounded). Several species have a rounder bulb and longer embolus, representing two or three lineages: the *floricola* group (*A. caricis*, *A. floricola*, *A. inexpectus*, *A. rupicola*, *A. sylvestris*), the *striatus* group (*A. striatus*, *A. rivalis*, *A. cutleri*, *A. dudkoi*) and the *zimmermanni* group (*A. zimmermanni*, *A. atricapillus*). These also have the folded spermathecae rotated slightly compared to the other *Attulus*, with the posterior lobe pointing posteriorly, rather than medially. The placement of *A. niveosignatus* in Attulus (Attulus) is somewhat doubtful, as the position of the tibial apophysis and the anterior medial epigynal openings both resemble those of *Sittisax* and Attulus subgenus Sittilong. We are reluctant to move it, however, until it is better studied.

Five species of Attulus (Attulus) are known from North America, all of which occur in Canada, as follows.

**Figures 15–30.**
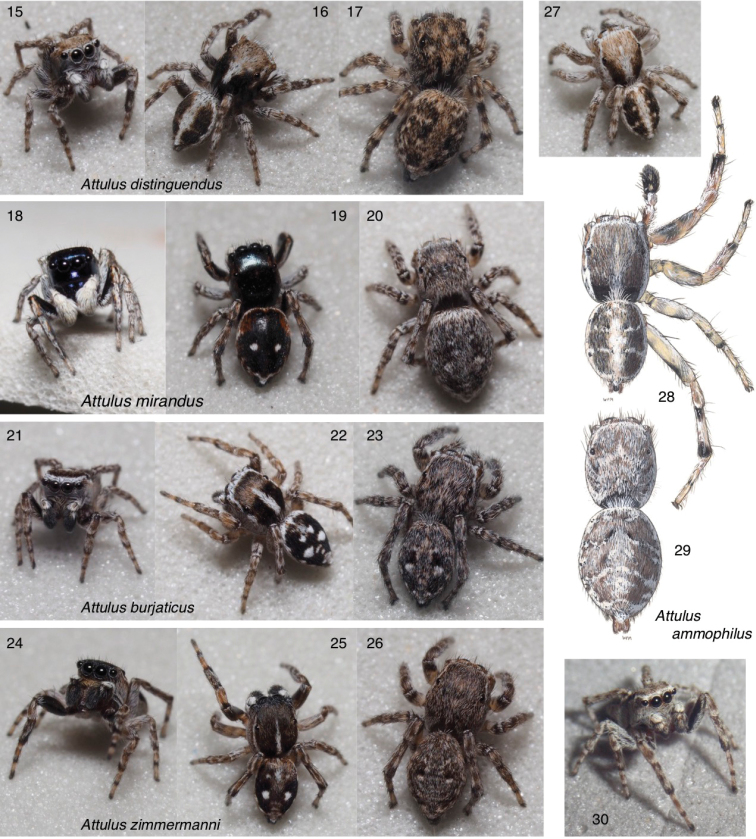
Attulus subgenus Attulus**15–17** male and female *A. distinguendus*, Tuva (50.746, 93.142) **18–20** male and female *A. mirandus*, Tuva (50.205, 95.135) **21–23***A. burjaticus*: **21** male, Tuva (50.68, 92.99) **22** male, Tuva (50.205, 95.135) **23** female, Tuva (50.68, 92.99) **24–26***A. zimmermanni*: **24, 25** male Novosibirsk Oblast (53.721, 77.726) **26** female Novosibirsk Oblast (53.730, 77.865) **27–30***A. ammophilus*: **27** male Tuva (50.6690, 92.9844) **28** male Ontario, Oakville **29** female Ontario, Hamilton **30** male British Columbia (49.08, -119.52). For additional images of *A. ammophilus*, see Figs 69–73. For additional images of Attulus (Attulus), see Figs 31–38, 49–73.

**Figures 31–38.**
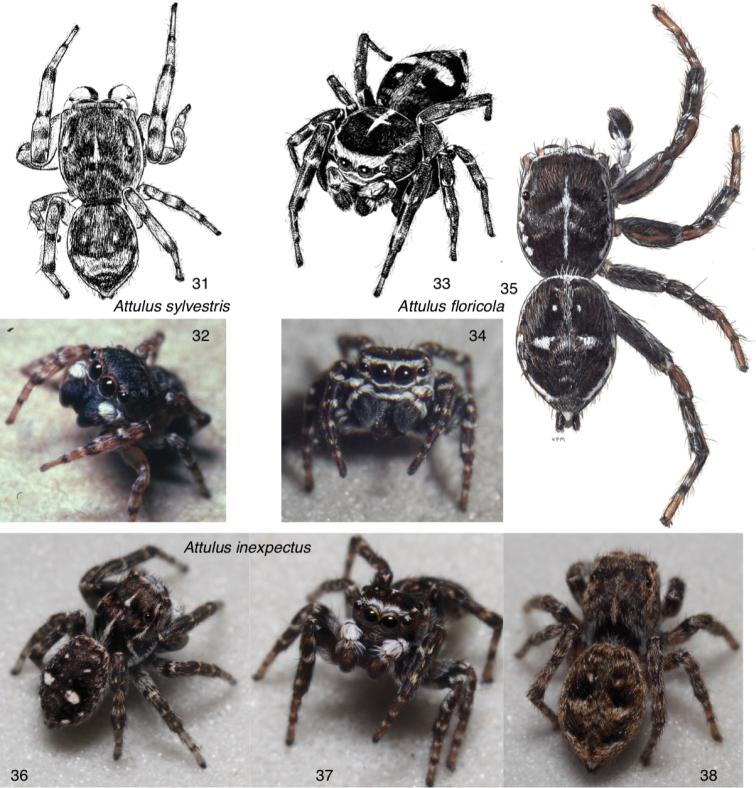
Attulus subgenus Attulus, continued (*floricola* group) **31, 32***Attulus sylvestris*: **31** male, Ontario, Ottawa **32** male, Maryland, Dorchester Co **33–35***A. floricola*: **33** male, Ontario, Port Cunnington **34** male, Ontario (46.9300, -79.7268) **35** male, Ontario, Gravenhurst **36–38***A. inexpectus*: **36, 37** male, Tuva (50.6690, 92.9844) **38** female, Tuva (51.316, 94.495). For additional images of the *floricola* group, see Figs 49–58.

####### Attulus (Attulus) ammophilus

Taxon classificationAnimaliaAraneaeSalticidae

(Thorell, 1875)

E4218DF4-2990-5E89-9397-AB35AB6A0B7F

Figures 27–30 , 69–73

Attus ammophilus Thorell, 1875

######## Remarks.

*Attulus ammophilus* is part of the species-rich *distinguendus* group that is otherwise unrepresented in North America. We have collected it from rocks on the ground in Ontario, British Columbia, and Utah, on litter among marsh plants along the edge of a lake in Siberia, and occasionally from buildings. It was introduced into North America during the 20^th^ century (Prószyński 1976, 1983).

######## Material examined

(all in UBC-SEM): Canada: Ontario: Hamilton (69 males, 35 females), Oakville (4 males, 3 females), Toronto (1 male), Windsor (1 male, 2 females); British Columbia: 49.7963, -119.5338 (1 male, 2 females), 49.95, -119.401 (3 males, 2 females); U.S.A.: Utah: 40.7482, -112.1856 (5 males, 7 females), 40.7672, -112.1575 (2 males).

####### Attulus (Attulus) floricola

Taxon classificationAnimaliaAraneaeSalticidae

(C.L. Koch, 1837)

9930DAE3-668A-59F5-BB00-CBC4BF493041

Figures 33–35 , 49–53

Euophrys floricola C. L. Koch, 1837.
Attus palustris Peckham & Peckham, 1883 (specimens in MCZ labelled as types, examined, but see below).
Attus morosus Banks, 1895 (synonymized by Prószyński 1980; confirmed here by examination of holotype female in MCZ from Olympia, Washington).

######## Remarks.

A widespread Holarctic species often found in retreats in dry flower heads in wetter areas such as marshes, *A. floricola* is distinctive for the sharp white lines around the eyes of males, forming an apparent mask (Fig. 34). *Attulus floricola* has often been confused in the past with its close relatives, but the distinctions have been clarified considerably by Prószyński (1980) and Logunov and Kronestedt (1997).

We treat the North American populations as full *floricola*, not a distinct subspecies. While Nearctic populations were long recognized as a separate species *palustris*, Prószyński (1980) suggested they are conspecific with the Eurasian populations. He maintained them as a distinct subspecies, but he expressed doubt as to whether even that distinction was warranted. We concur with his skepticism. If any consistent differences exist between the continents, they are no more visible than any differences that might exist between the Eurasian and North American populations of other species for which we don’t recognize subspecies such as *Sittisax ranieri*, *Attulus cutleri*, *Dendryphantes nigromaculatus* (Keyserling, 1885), *Pellenes ignifrons* (Grube, 1861), and *Pellenes lapponicus* (Sundevall, 1833).

The results of our COI analysis of Palearctic and Nearctic *floricola* group (Fig. 104) show all *floricola* to be close on the gene tree, with the New World specimens in two clades (not clearly related to one another) and the German specimens in a third clade. This suggests that *A. floricola* is not cleanly or deeply divided between the Nearctic and Palearctic. The molecular and morphological evidence leads us to fully synonymize *palustris* into *floricola*.

Within North America, the characterization of *A. floricola* has been muddied by confusion with a second species, *A. sylvestris*. *Attulus sylvestris*, long synonymized with *palustris*, is a distinctively different species. *Attulus floricola* is larger-bodied, has a much more contrasting colour pattern, and longer legs. *Attulus floricola* has a different angle of the spermaphore loop (subtle but consistent; Fig. 49 vs. Fig. 54), and in females the darkness of the spermathecal lobe is visible through the anteriormost portion of the epigynal atrium (Fig. 50 vs. Fig. 55). *Attulus sylvestris* has genitalia more similar to those of the Eurasian *A. caricis*, *A. rupicola*, and *A. inexpectus*, as noted below. The synonymy of *sylvestris* with *palustris* was originally proposed by Peckham and Peckham (1909), after which Kaston (1948) may have stirred confusion by choosing to illustrate *palustris* using Emerton’s (1891) figure of *sylvestris*.

A more serious confusion apparently occurred with the labelling of type specimens of *Attus palustris*. The description by Peckham and Peckham (1883) refers without doubt to the common white-striped species long known as *Sitticus palustris* (Fig. 34): males dark brown, reddish toward eyes, marked with white lines, including those around the eyes, and palp with some white hairs on several segments of the palp. As well, the habitat suggested by the name “*palustris*” is marsh or swamp, more typical for *A. floricola* than *A. sylvestris*. However, the specimens labelled as the types of *Attus palustris* in the MCZ are clearly specimens of the less common dusty brown species (i.e., Emerton’s *sylvestris*, Fig. 32). These specimens, we argue, are mislabelled: they do not match the Peckhams’ description, and thus are not the type specimens of *A. palustris*. That the Peckhams viewed the white-striped form as typical *palustris* can be judged not only from their 1883 description, but also from their implicitly distinguishing two forms in their 1909 statement “Mr. Emerton agrees with us that the form which he described as *sylvestris* is a variety of *palustris*, with the leg a little shorter and stouter.” The label of the holotype does not appear to be in the handwriting of either George or Elizabeth Peckham, and it is possible that these “types” were so labelled after 1883.

At stake is not the name used for the common white-striped species (which would be *floricola* regardless), but the name for the uncommon dusty brown species, which would be *palustris* were we to accept these specimens as its types. However, as argued above, they are not the types. We therefore treat *palustris* as a synonym of *floricola*, and *sylvestris* as the name for the dusty brown species. To settle the mislabelling properly, a male specimen of the white-striped species from Wisconsin (the type locality) should be designated as the neotype or lectotype of *palustris*. We have not yet done so as we await reexamination of the full Peckham collection in case specimens can be located that might be identifiable as from the true type series.

######## Material examined.

Canada: British Columbia: Richmond (2 females), 49.66, -114.73 (1 female), 49.45, -115.08 (3 males, 6 females); Alberta: 52.46, -113.94 (1 male); Ontario: Richmond (2 males, 1 female), Gravenhurst (3 males), Port Cunnington (1 female); Dwight (2 males, 5 females), Batchawana Bay (1 female), Woodstock (3 females), 46.9300, -79.7268 (2 males, 1 female), 42.53, -80.12 (1 female), 43.2626, -80.5636 (1 male), 49.0852, -81.3237 (1 female); Quebec: Touraine (1 male); Nova ScotiA: 44.4318, -64.6075 (1 male); U.S.A.: Washington: 46.43, -123.86 (2 males); Colorado: Jackson Lake State Rec. Area (1 male); Nebraska: 41.88, -103.09 (1 female).

####### Attulus (Attulus) sylvestris

Taxon classificationAnimaliaAraneaeSalticidae

(Emerton, 1891), restored (removed from synonymy with S. floricola)

76CB4AB2-16AC-5804-AD07-1211A59B74C5

Figures 31 , 32 , 54–58

Attus sylvestris Emerton, 1891 (Holotype male in MCZ from Beverly, Massachusetts, examined).
Sitticus magnus Chamberlin & Ivie, 1944, syn. nov.
Sitticus rupicola – Prószyński, 1980, figs 58, 59 (misidentification), specimen from Texas.

######## Remarks.

A widespread but little-known Nearctic species, *A. sylvestris* can be found on partially shaded ground where the males stand out for their tiny bouncing bright white spots (the white tuft of setae on the palp’s tibia). We have found them on rocks and leaf litter along a forest edge in Ontario, on the ground at the edge of a creek in a forest in California, and on forest leaf litter in Maryland. See discussion under *A. floricola* about why we judge *A. sylvestris* to be the proper name of this species, at issue because of confusion over the type specimens of *Attus palustris*.

Both males and females have shorter legs and less contrasting markings than in *A. floricola*, but the distinction of markings is most notable in the male, which lacks the high-contrast white stripes on dark brown seen in *A. floricola*. The white setae on the male’s palp are concentrated on just the tibia and end of the femur. The bulb of the palp is rotated slightly more than in *A. floricola*, and thus the spermophore’s path shows an upturn (i.e., the loop is angled to point distally instead of basally as in *floricola*), and the female’s copulatory ducts arrive further to the posterior before looping back anteriorly to enter the spermathecae. In these regards the genitalia resemble those of the Eurasian *A. rupicola*, *A. caricis*, and *A. inexpectus* (Logunov and Kronsestedt 1997). *Attulus sylvestris* is most similar to *A. caricis* in appearance (low-contrast brown markings), in having a small loop of the copulatory duct, and small body size, but differs in brighter markings on the palp, a more anteriorly-placed junction where the ducts enter the spermatheca, a larger epigynal RTA coupling pocket, and a more distinctly swollen bulb of the spermatheca. (They are also distinct on the COI tree, Fig. 104.) The synonymy of *magnus* can be determined by its original description and Prószyński’s (1980) excellent drawing of the vulva of the holotype female. The female from Texas tentatively identified by Prószyński (1980: 15, figs 58, 59) as *S. rupicola* is considered here to be *S. sylvestris* based on his clear drawings showing the loop of the copulatory duct slightly bigger than typical, but not reaching nearly as far to the posterior as in *S. rupicola*.

######## Material examined

(all in UBC-SEM except as indicated): Canada: Ontario: Ottawa, Britannia Bay, 45.374, -75.796 (26 males, 3 females), Long Point, 42.53, -80.12 (2 females); U.S.A.: Maryland: Dorchester Co. (1 male 1 female, MCZ); Colorado: Morgan Co., Fort Morgan (1 female); California: Smith Redwoods State Reserve (1 male), 36.3907, -121.5951 (2 females), 36.3742, -121.5614 (1 male, 4 females).

####### Attulus (Attulus) striatus

Taxon classificationAnimaliaAraneaeSalticidae

(Emerton, 1911)

AAC02BF0-0E0B-5E7A-AB7A-40E892233E56

Figures 59–63

Sitticus striatus Emerton, 1911

######## Remarks.

*Attulus striatus* is a small-bodied Northern species with distinctively striped males, from sphagnum bogs. Although we were unable to obtain molecular data for it or the similar *A. rivalis* and *A. cutleri*, these three species can be placed into subgenus Attulus with some confidence, based on their boxy carapaces (resembling the other Attulus (Attulus) rather than Attulus (Sitticus)), and the genitalic similarities with subgenus Attulus, including the two small posterior openings of the epigyne on either side of a narrow triangular RTA coupling pocket. Prószyński (1980) considered them close to the *floricola* group in particular.

We reinstate *S. rivalis* Simon, 1937 as a distinct species (contra Prószyński 2017a), accepting Logunov’s (2004) clear evidence for their distinction (primarily, in the rotation of the bulb of the palp). *Attulus rivalis* is known from France, also from sphagnum bogs.

######## Material examined

(all UBC-SEM): Canada: Alberta: S. Islay (3 female), Beaverhill Lake (1 female); Ontario: 48.3260, -76.8365 (1 female); 3 km S. Richmond (6 males, 2 females); New Brunswick: Chipman (1 male, 1 female); U.S.A.: New Hampshire: Ponemah Bog (1 female).

####### Attulus (Attulus) cutleri

Taxon classificationAnimaliaAraneaeSalticidae

(Prószyński, 1980)

5E5F9845-3A33-5AFA-9D64-B84BF9E2707E

Figures 64–68

Sitticus cutleri Prószyński, 1980
Sitticus gertschi Prószyński, 1980

######## Remarks.

A Sibero-American boreal species that is little collected, resembling closely *A. striatus* but differing in having less striped legs, a less rotated bulb of the male palp, more medially placed epigynal openings. Collected on “leaf litter under small *Salix* just above stream” (D. Maddison, June 1981, Inuvik).

######## Material examined.

Canada: Northwest Territories: Wrigley (1 female, CNC), Inuvik (1 male, UBC-SEM).

###### Subgenus Sitticus Simon, 1901

Figures 39–47, 74–88

*Sitticus* Simon, 1901 (type species *Araneus terebratus* Clerck 1757)

*Hypositticus* Lohmander, 1944 (type species *Aranea pubescens* Fabricius, 1775)

*Sittipub* Prószyński, 2016 (type species *Aranea pubescens* Fabricius, 1775)

**Figures 39–47.**
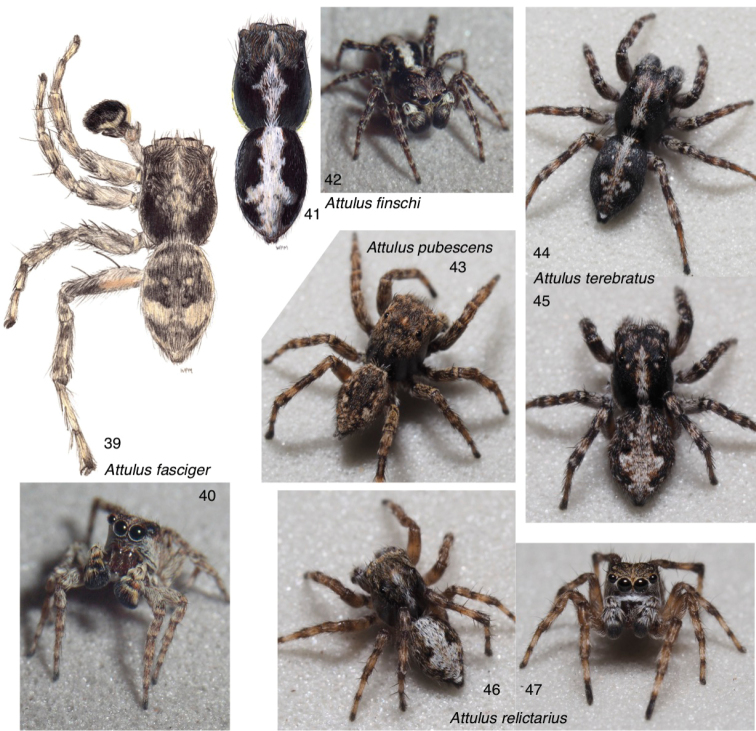
Attulus subgenus Sitticus**39, 40***A. fasciger*, male, Ontario (43.3508, -79.7593) **41, 42***A. finschi*: **41** male, Saskatchewan (55.31, -105.11) **42** male body, Ontario, 4 miles S of Wawa **44, 45***A. terebratus*: **44** male, Novosibirsk Oblast (53.730, 77.865) **45** female, Novosibirsk **46, 47***A. relictarius* male, Stavropol Krai, (43.88, 42.70). For additional images of Attulus (Sitticus), see Figs 74–88.

The species placed here, despite having palpi with very different embolus lengths, share a similarly narrow and high body with relatively long legs (Figs 39–47), and (except for *A. relictarius*) a dramatically large RTA, broadly arising from the tibia and sweeping diagonally to the retrolateral and distal (Figs 74, 79, 84). Several species have a long embolus and correspondingly long and convoluted copulatory ducts, though *A. pubescens* and *A. relictarius* have among the shortest in sitticines. The species of *Sitticus* are Palearctic or Holarctic; the following three are found in Canada.

**Figure 48.**
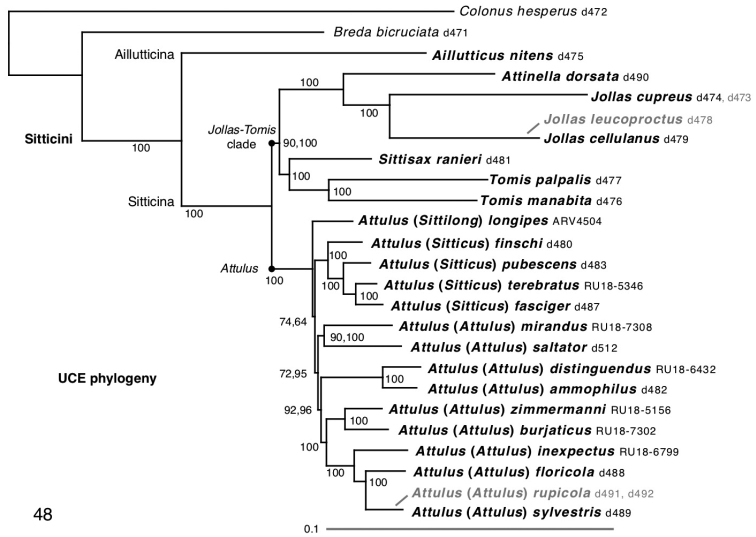
Maximum likelihood phylogeny from 757 concatenated UCE loci (average 113231 base pairs/taxon) analyzed primarily for the 23 Core Taxa in black (IQ-TREE, partitioned by locus). Topology is identical in unpartitioned analyses, with nearly identical branch lengths. Bootstrap percentage values from 1000 replicates shown for each clade. Where two numbers are shown, the first is the bootstrap percentage for the partitioned analysis, the second for the unpartitioned analysis. Where one number is shown, both analyses yielded the same percentage. An analysis of the All Taxa dataset, including the weakly-sequenced taxa in grey, yielded the same topology.

**Figures 49–68.**
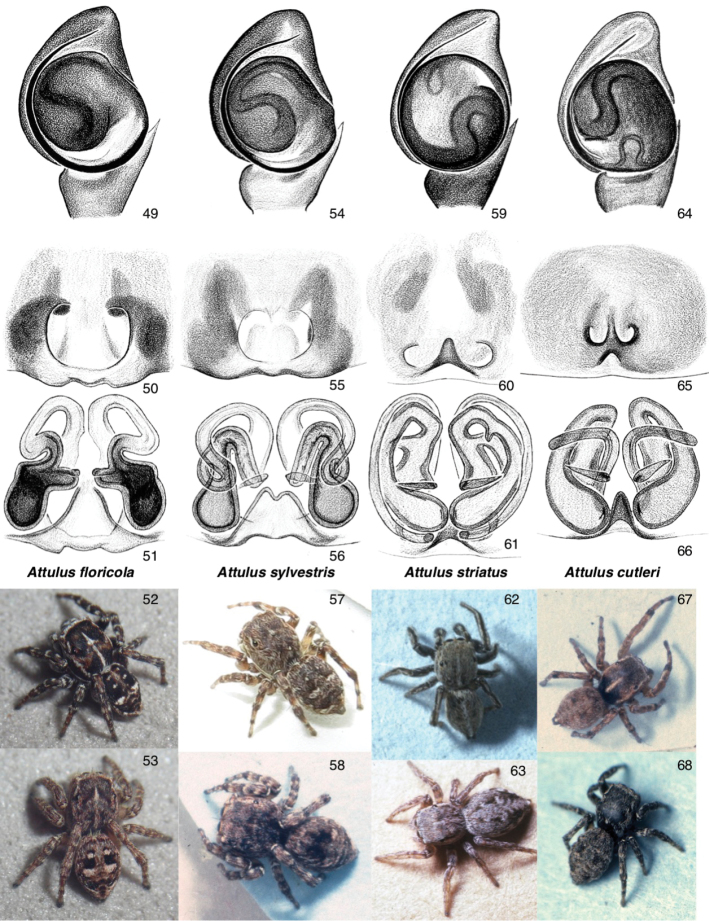
Sitticines of Canada: Attulus subgenus Attulus (for *A. ammophilus*, see Figs 69–73) **49–53***Attulus floricola*: **49** palp (Ontario, Gravenhurst) **50, 51** ventral view of epigyne, dorsal view of cleared vulva (Ontario, Gravenhurst) **52** male (Ontario, 46.9300, -79.7268) **53** female (Ontario, 46.9300, -79.7268) **54–58***Attulus sylvestris*: **54** palp (Ontario, Ottawa) **55, 56** ventral view of epigyne, dorsal view of cleared vulva (Ontario, Ottawa) **57** male (California, 36.3646, -121.5544) **58** female (Ontario, 42.55, -80.13) **59–63***Attulus striatus*: **59** palp (Ontario, 45.1453, -75.8467) **60, 61** ventral view of epigyne, dorsal view of cleared vulva (Ontario, 45.1453, -75.8467) **62** male (Ontario, 45.1453, -75.8467) **63** female (New Hampshire, Ponemah Bog) **64–68***Attulus cutleri*: **64** palp (Northwest Territories, Wrigley) **65, 66** ventral view of epigyne, dorsal view of cleared vulva (Northwest Territories, Wrigley) **67** male (Northwest Territories, Inuvik) **68** female (Yukon, 67.57, -139.67). For habitus of other *Attulus* species, see Figs 15–38.

####### Attulus (Sitticus) finschi

Taxon classificationAnimaliaAraneaeSalticidae

(L. Koch, 1879)

A6B6535C-374E-5678-A043-46B18B092BA8

Figures 41 , 42 , 79–83

Attus finschii L. Koch, 1879
Euophrys cruciatus Emerton, 1891

######## Remarks.

The natty contrasting black-and-white markings distinguish *Attulus finschi* from the closely related *A. fasciger*. *Attulus finschi* is the only *Sitticus* that has likely been in the Americas for thousands of years; it also lives in Siberia. It is found in boreal habitats on tree trunks.

######## Material examined

(all UBC-SEM): Canada: Saskatchewan: 55.31, -105.11 (1 male, 1 female), 55.27, -105.19 (1 female); Ontario: Wawa (1 male), Nipigon (1 female), 48.9143, -80.9446 (2 females); New Brunswick: Doaktown (1 male).

####### Attulus (Sitticus) fasciger

Taxon classificationAnimaliaAraneaeSalticidae

(Simon, 1880)

6DABB58A-A64F-527A-991B-2DBE3FC5CB77

Figures 39 , 40 , 74–78

Attus fasciger Simon, 1880

######## Remarks.

This species, introduced to North America apparently in the middle of the 20^th^ century (Cutler 1990), is typically found on buildings. The large male palp and spaghetti-like copulatory ducts distinguish it from other species in North America except the differently-coloured *A. finschi*.

######## Material examined

(all in UBC-SEM): Canada: Ontario: Burlington (3 males, 6 females), 43.35074, -79.75928 (25 males, 14 females); U.S.A.: Missouri: Dogtown (3 males, 4 females); Massachusetts: Cambridge (1 female).

####### Attulus (Sitticus) pubescens

Taxon classificationAnimaliaAraneaeSalticidae

(Fabricius, 1775)

769ED5BD-20F4-548B-B403-2CEBC34085F1

Figures 43 , 84–88

Aranea pubescens Fabricius, 1775

######## Remarks.

Although closely related to *A. fasciger* and *A. terebratus*, which have among the longest emboli and copulatory ducts in sitticines, *Attulus pubescens* has among the shortest known in sitticines. The very large RTA is distinctive. Introduced to North America in the 20^th^ century (Cutler 1990).

######## Material examined

(All in UBC-SEM): Canada: British Columbia: Vancouver (1 male 1 female); U.S.A.: Massachusetts: Cambridge (3 males, 3 females), Boston (2 males), Milton (2 males), Arlington (1 female).

**Figures 69–88.**
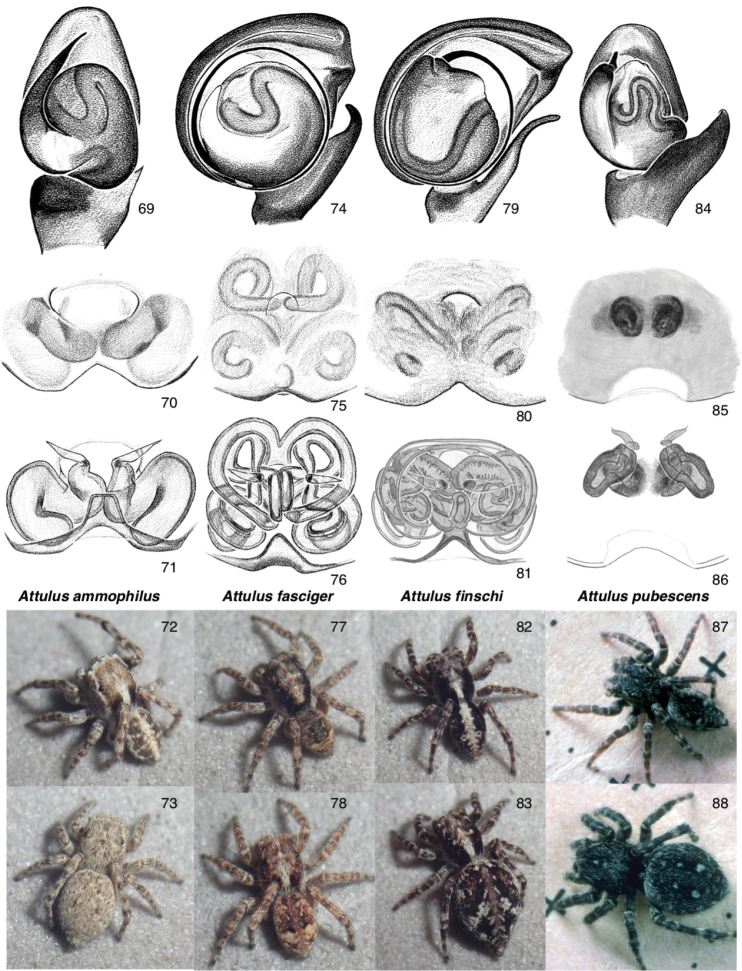
Sitticines of Canada: *Attulus*, continued **69–73**Attulus (Attulus) ammophilus: **69**palp (Ontario, Oakville) **70, 71** ventral view of epigyne, dorsal view of cleared vulva (Ontario, Hamilton) **72** male (British Columbia, 49.08, -119.52) **73** female (British Columbia, 49.08, -119.52) **74–78**A. (Sitticus) fasciger (Ontario, 43.3508, -79.7593): **74** palp **75, 76** ventral view of epigyne, dorsal view of cleared vulva **77** male **78** female **79–83**A. (S.) finschi: **79** palp (Ontario, Wawa) **80, 81** ventral view of epigyne, dorsal view of cleared vulva (Saskatchewan, 55.31, -105.11) **82** male (Saskatchewan, 55.31, -105.11) **83** female (Saskatchewan, 55.27, -105.19) **84–88**A. (S.) pubescens: **84** palp (Massachusetts, Milton) **85, 86** ventral view of epigyne, dorsal view of cleared vulva (Massachusetts, Arlington) **87** male (Massachusetts, Cambridge) **88** female (Massachusetts, Cambridge). For other images of Attulus (Sitticus), see Figs 39–47.

###### Subgenus Sittilong Prószyński, 2017

*Sittilong* Prószyński, 2017 (type species *Attus longipes* Canestrini, 1873)

The single species Attulus (Sittilong) longipes of the European Alps is peculiar for its flat body and long first legs in the male, as well as its genitalia. Like *Sittisax* and other members of the *Jollas*-*Tomis* clade, the RTA is offset basally, and the epigynal openings are anterior and medial. The little-studied *Attulus niveosignatus* has somewhat similar genitalia and may also belong in *Sittilong*.

##### The *Jollas*-*Tomis* clade

We have chosen not to subdivide the Neotropical Sitticina more finely than into two genera, *Tomis* and *Jollas*, primarily because the fauna is poorly enough known that it is as yet unclear what coarseness of division would be most useful. We might have synonymized their respective Nearctic offshoots (*Sittisax* into *Tomis*, and *Attinella* into *Jollas*), but by retaining them as distinct, we facilitate the eventual splitting of both *Tomis* and *Jollas* as their species become better known. We choose splitting in the *Jollas*-*Tomis* clade, in contrast to lumping with *Attulus*, because the phylogenetic divergences are so much deeper in the former compared to the latter.

The *Jollas*-*Tomis* clade includes four genera with 31 species:

*Attinella* Banks, 1905, with three species:

*Attinella concolor* (Banks, 1895), comb. nov., transferred from *Sitticus*

*Attinella dorsata* (Banks, 1895), combination restored, transferred from *Sitticus* (type species)

*Attinella juniperi* (Gertsch & Riechert, 1976), comb. nov., transferred from *Sittiab*

*Jollas* Simon, 1901, with 12 species:

*Jollas amazonicus* Galiano, 1991

*Jollas cellulanus* (Galiano, 1989), comb. nov., transferred from *Sitticus*

*Jollas cupreus* W. Maddison, sp. nov.

*Jollas flabellatus* (Galiano, 1989), comb. nov., transferred from *Sitticus*

*Jollas geniculatus* Simon, 1901 (type species)

*Jollas hawkeswoodi* Makhan, 2007

*Jollas leucoproctus* (Mello-Leitão, 1944), comb. nov., transferred from *Sitticus*

*Jollas manantiales* Galiano, 1991

*Jollas paranacito* Galiano, 1991

*Jollas pompatus* (Peckham & Peckham, 1894)

*Jollas puntalara* Galiano, 1991

*Jollas richardwellsi* Makhan, 2009

*Sittisax* Prószyński, 2017, with two species:

*Sittisax ranieri* (Peckham & Peckham, 1909)

*Sittisax saxicola* (C. L. Koch, 1846) (type species)

*Tomis* F.O. Pickard-Cambridge, 1901, with 14 species

*Tomis canus* Galiano, 1977, combination restored, transferred from *Sitticus*

*Tomis kratochvili* (Caporiacco, 1947), comb. nov., transferred from *Pseudattulus*

*Tomis manabita* W. Maddison, sp. nov.

*Tomis mazorcanus* (Chamberlin, 1920), comb. nov., transferred from *Sitticus*

*Tomis mona* (Bryant, 1947), comb. nov., transferred from *Sidusa*

*Tomis palpalis* F. O. Pickard-Cambridge, 1901, combination restored, transferred from *Sitticus* (type species)

*Tomis pavidus* (Bryant, 1942), comb. nov., transferred from *Sidusa*

*Tomis phaleratus* (Galiano & Baert, 1990), comb. nov., transferred from *Sitticus*

*Tomis pintanus* (Edwards & Baert, 2018, comb. nov., transferred from *Sitticus*

*Tomis tenebricus* (Galiano & Baert, 1990), comb. nov., transferred from *Sitticus*

*Tomis trisetosus* (Edwards & Baert, 2018), comb. nov., transferred from *Sitticus*

*Tomis uber* (Galiano & Baert, 1990), comb. nov., transferred from *Sitticus*

*Tomis vanvolsemorum* (Baert, 2011), comb. nov., transferred from *Sitticus*

*Tomis welchi* (Gertsch & Mulaik, 1936), comb. nov., transferred from *Sitticus*

##### Genus *Attinella* Banks, 1905, restored (removed from synonymy with *Sitticus*)

*Attinella* Banks, 1905 (type species *Attus dorsatus* Banks, 1895)

*Sittiab* Prószyński, 2017 (type species *Sitticus absolutus* Gertsch & Mulaik, 1936), syn. nov.

Small species from southern North America, related to the *Jollas* of South America. Except for the thin longitudinal stripes of *A. dorsata*, their bodies are more or less unmarked. Like many other members of the *Jollas*-*Tomis* clade, the RTA is long and thin, paralleling the axis of the palp, the tibia is robust, and the embolus is fairly short. The first leg’s trochanter is unusually long in at least some males (note angles in Fig. 12), though less so than in *Jollas*. The epigynal openings are anterior, with the ducts (intially fused) leading to the posterior and to fairly small spermathecae. As noted below under *A. dorsata*, the synonymy of *Sitticus absolutus* with *Attus dorsatus* leads to *Sittiab* being a junior synonym of *Attinella*. Two species of *Attinella* reach Canada.

###### Attinella concolor

Taxon classificationAnimaliaAraneaeSalticidae

(Banks, 1895)

17D19A81-6E3D-5A0F-96B9-320243AB7029

Figures 89–93

Attus concolor Banks, 1895 (holotype examined; see Maddison 1996: 270)
Sittacus cursor Barrows, 1919, synonymy restored
Sitticus floridanus Gertsch & Mulaik, 1936

####### Remarks.

A small unmarked leaf litter species, known best from the southeastern United States, but recently reported from Canada in the BOLD barcode database (Ratnasingam and Hebert 2007, 2013), from the extreme southern point in Ontario (Point Pelee National Park, specimens PPELE142-11, PPELE183-11, CNPPE2332-12, PPELE666-11, PPELE644-11).

Prószyński (2017a) rejected Maddison’s (1996) synonymy of *cursor* with *concolor* on the basis of “lack of documentation”, an extra specimen inside the type vial, and the fact that it was published in a revision of *Pelegrina*. However, Maddison (1996) indicated clearly the evidence that identified the holotype within the vial (by its location, labeling, and match to Banks’s description), and the features that matched the specimen to *Sittacus cursor* Barrows; that the nomenclatural act was published in a revision of a different salticid genus has no bearing on the validity of the act. Maddison’s synonymy, therefore, is reaffirmed here as valid.

####### Material examined.

U.S.A.: Florida: Gainesville (1 male, 1 female, UBC-SEM).

**Figures 89–103.**
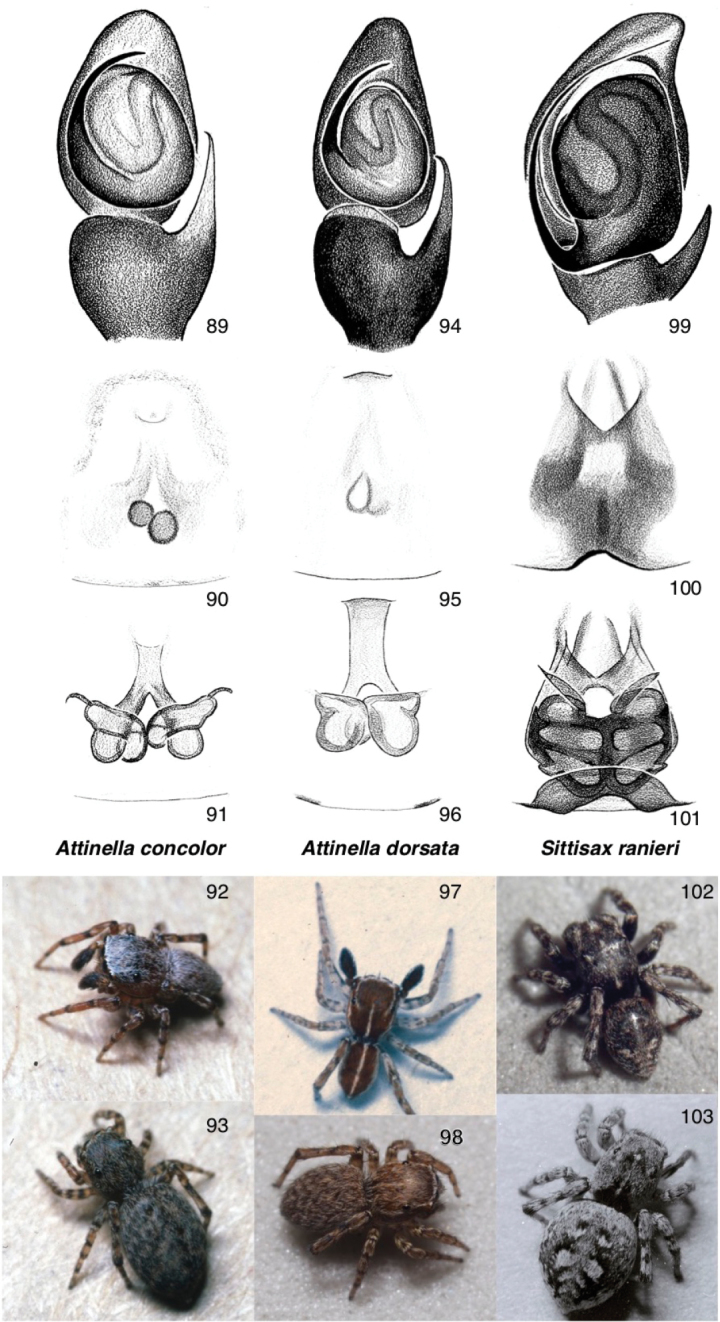
Sitticines of Canada: the *Jollas*-*Tomis* clade, represented by the genera *Attinella* and *Sittisax***89–93***Attinella concolor*: **89** palp (Florida, Gainesville) **90, 91** ventral view of epigyne, dorsal view of cleared vulva (Florida, Gainesville) **92** male (Texas, 30.10, -97.25) **93** female (Texas, 30.10, -97.25) **94–98***Attinella dorsata*: **94** palp (California, San Diego County) **95, 96** ventral view of epigyne, dorsal view of cleared vulva (British Columbia, Nanaimo) **97** male (California, Siskiyou County) **98** female (British Columbia, 48.870, -123.379) **99–103***Sittisax ranieri*: **99** palp (Northwest Territories, Tuktoyaktuk) **100, 101** ventral view of epigyne, dorsal view of cleared vulva (Nunavut, Baffin Island) **102** male (Saskatchewan, 55.27, -105.19) **103** female (Ontario, Old Woman Bay).

**Figure 104.**
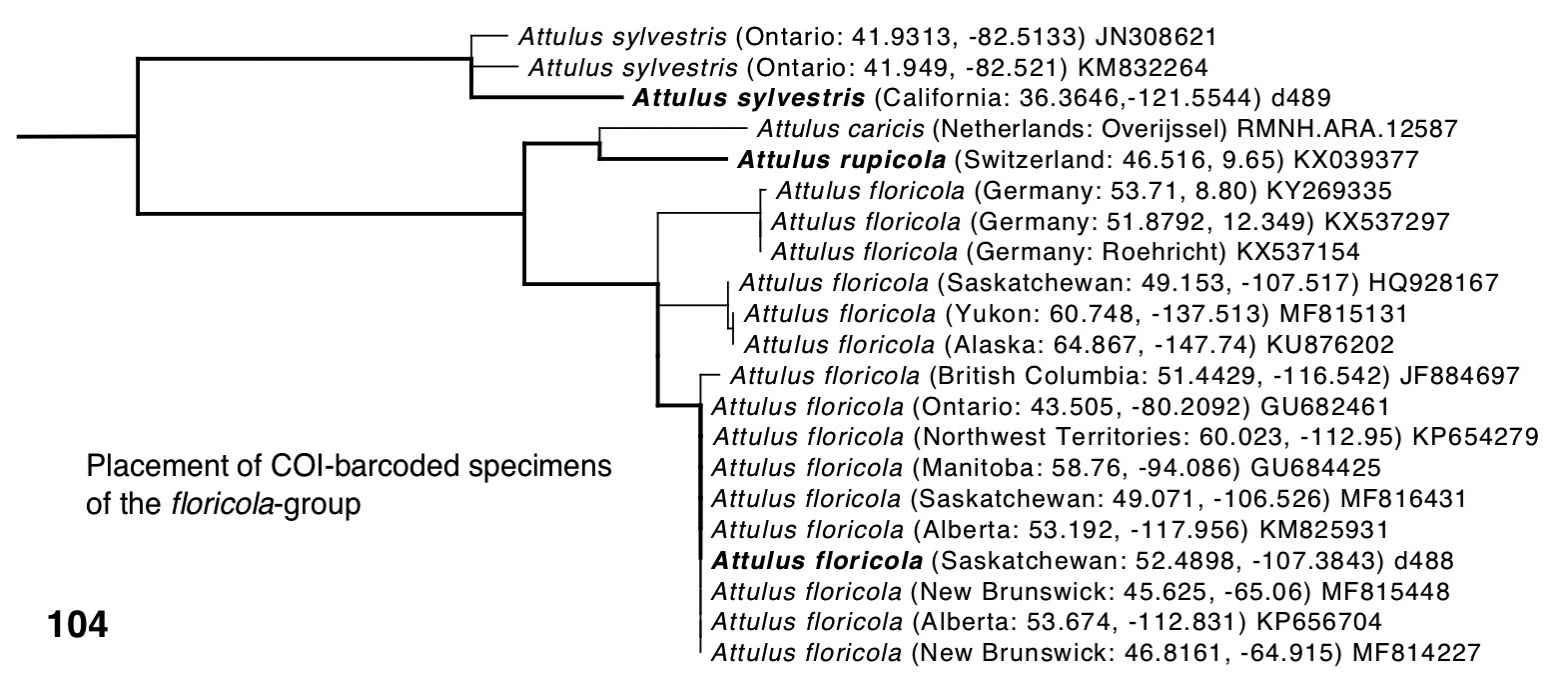
Relationships among *Attulus floricola* mitochondrial COI sequences in the context of the *floricola* group. Specimens in bold had their relationships constrained by the UCE phylogeny of Fig. 48; not shown are the relationships outside the *floricola* group, which are fixed to match the UCE phylogeny. The placement of non-bold specimens on this constrained skeletal tree was inferred by maximum likelihood (RAxML, codon positions as separate partitions).

###### Attinella dorsata

Taxon classificationAnimaliaAraneaeSalticidae

(Banks, 1895)

41DCF2E7-18C0-593D-A4B5-E528627B081E

Figures 11–14 , 94–98 , 105

Attus dorsatus Banks, 1895 (holotype female in MCZ from California: Los Angeles, examined)
Sitticus absolutus Gertsch & Mulaik, 1936, synonymy restored
Sitticus callidus Gertsch & Mulaik, 1936, synonymy restored

####### Remarks.

**Figures 105–107.**
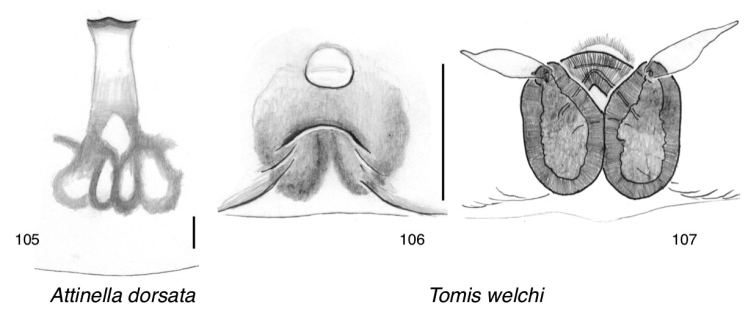
Epigynes of *Attinella dorsata* and *Tomis welchi***105** holotype of *Attus dorsatus* Banks, 1895, epigyne, ventral view **106, 107** holotype of *Sitticus welchi* Gertsch & Mulaik, 1936 **106** epigyne, ventral view **107** cleared vulva, dorsal view.

While females of this small Southwestern desert-dwelling species are indistinctly unmarked, males tend to be reddish with a narrow central longitudinal stripe (Figs 11–14). Prószyński (2017a) rejected Richman’s (1979) synonymy of *Attinella dorsata* (Banks, 1895) with *Sitticus absolutus*, saying that *dorsata* is unidentifiable. That statement is false, given that the type specimen is in the MCZ and in good condition. The specimen (examined) has a relatively wide carapace with single thin longitudinal pale line dorsally, long fourth leg, no retromarginal cheliceral tooth, and epigyne (Fig. 105) with a single anterior opening that leads posteriorly through a single duct that splits before the spermathecae, which are visible as two small medial pear-shapes flanked by slightly larger chambers. In these respects, it clearly falls within our current concept of *Sitticus absolutus* as a common, widespread, and relatively uniform species from Texas to California north to Canada (see illustrations by Gertsch and Mulaik 1936, Prószyński 1973). Even if future work were to show that the Californian populations (type locality of *dorsatus*) and Texan populations (type locality of *absolutus*) represent distinct species, they are extremely closely related, certainly congeneric. *Attus dorsatus* is a member of these Californian populations, and for this reason the synonymy of *Sittiab* (type species *Sitticus absolutus*) with *Attinella* (type species *Attus dorsatus*) is assured.

####### Material examined.

Canada: British Columbia: Summerland (1 male, CNC), Galiano Island (2 males, 3 females, UBC-SEM), Nanaimo (1 female). U.S.A.: CALIFORNIA: Humboldt Co., Orleans (1 male, UBC-SEM), Siskyou Co., Beaver Creek and Klamath River (1 male, UBC-SEM), San Diego Co., Johnson Canyon (1 male 1 female, UBC-SEM), El Dorado Co., Camino (1 female, UBC-SEM), Inyo Co., Gilbert Summit (1 female, UBC-SEM); Utah: Millard Co., Sevier Lake (1 male, UBC-SEM); Colorado: Morgan Co., Jackson Lake (1 male, UBC-SEM), Jefferson Co., Golden (2 females, UBC-SEM); Texas: Jim Hogg Co., Guerra (1 female, UBC-SEM), Pecos Co., Fort Stockton (1 female, UBC-SEM).

##### Genus *Jollas* Simon, 1901

Figures 7–10, 108–119

*Jollas* Simon, 1901 (type species *Jollas geniculatus* Simon, 1901)

*Oningis* Simon, 1901 (type species *Neon pompatus* Peckham & Peckham, 1893)

A Neotropical group, consisting of two species groups, the small glabrous or shiny *geniculatus* group (Galiano 1991b), and the typically grey *leucoproctus* group (Galiano 1989). The male’s first trochanter is relatively long, approximately as long as the coxa (Galiano 1989). Typically, the male’s first tibia and patella are marked by dark lines on the prolateral face. Epigynal openings are medial, with ducts proceeding toward the lateral. Most species have a long thin RTA, though that is also seen in many *Tomis* and *Attinella*.

**Figures 108–119.**
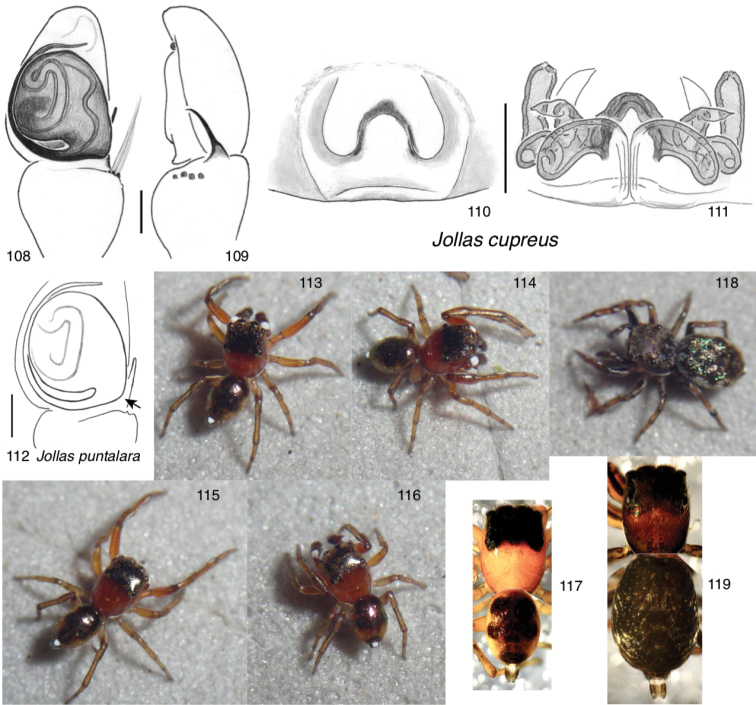
*Jollas cupreus*, sp. nov. (except **112**, *J. puntalara*) **108, 109** Left palp of holotype **108** ventral view **109** retrolateral view **110** ventral view of epigyne of paratype **111** dorsal view of same, cleared **112** palp of holotype of *J. puntalara* Galiano **113–115** holotype male **116** male from Yasuní, Ecuador (-0.675, -76.397) **117** holotype male in alcohol **118, 119** paratype female.

###### Jollas cupreus

Taxon classificationAnimaliaAraneaeSalticidae

W. Maddison sp. nov.

E4B7CD04-26B9-534B-9A21-FC95631A44C0

http://zoobank.org/68F87DD6-8C31-4D0B-A349-245B9B201CF3

Figures 7 , 108–111 , 113–119

####### Type material.

Male holotype and 2 male, 3 female paratypes from Ecuador: Orellana: Río Bigal Reserve, main camp area. 0.5251, -77.4177. 950 m elev. 1–5 November 2010. W & D Maddison, M Vega, M Reyes. WPM#10-041c. The holotype (specimen ECU2010-2060) pertains to the Museum of Zoology, Pontificia Universidad Católica, Quito, Ecuador (QCAZ), but is currently held in the Spencer Entomological Collection at the Beaty Biodiversity Museum, University of British Columbia (UBC-SEM).

####### Etymology.

Refers to the copper colour of males.

A species common in eastern Ecuador on disturbed open grassy ground. It was used in the molecular phylogenetic study of Maddison and Hedin (2003) under the name “*Jollas* sp.” (voucher S162) from Sucumbios, Ecuador.

####### Diagnosis.

Differs from the very similar *Jollas puntalara* Galiano, 1991 in the thinner and straighter RTA and the angle at which the embolus arises. The RTA is more or less straight until a curl at the tip, but it narrows dramatically for its terminal three quarters (Fig. 109), whereas in *J. puntalara* (Galiano, 1991b: fig. 26) it bends at the midpoint and thins much less dramatically. The embolus of *J. cupreus*, as it arises, proceeds directly to the prolateral, thus generating an angle in the retrolateral-basal corner of the bulb (like a chin pointing to the retrolateral), while the embolus of *J. puntalara* emerges angled toward the basal, leaving the bulb more rounded (arrow in Fig. 112). These differences are small but consistent, insofar as all Ecuadorian specimens show the distinct “chin” at the base of the embolus and the narrower RTA. It might usually be conservative to leave such close forms as a single species, but given that there is considerable data (molecular phylogenetic and chromosome) attached to the Ecuadorian form, it is safer to name it and thus provide an unambiguous anchor for these data. (Cristian Grismado kindly supplied photographs of Galiano’s (1991b) holotype of *Jollas puntalara* to facilitate our comparison, although these differences can be seen as well in her figures 26 and 29.)

####### Description.

**Male** (holotype). Carapace length 1.37; abdomen length 1.16. ***Carapace*** orange with a black ocular area, mostly glabrous, with only a few scattered setae. ***Clypeus*** orange-brown. ***Chelicerae*** vertical, orange. ***Palp*** orange-brown except for dark brown cymbium, with dark setae except brilliant white patch of setae dorsally on patella. ***Legs*** long, especially the first and fourth. Legs honey coloured to orange-brown except for a strong black line on prolateral-ventral face of first patella, tibia and metatarsus. ***Embolus*** arises at ca. 5 o’clock and curls half-way around bulb. Tibia somewhat bulbous, broad, with bases of setae on retrolateral side forming row of tubercles. RTA begins broad but then narrows abruptly at ca. one quarter its length, from which point it proceeds straight until just before the tip, where it curls. ***Abdomen*** orange-brown, with black scalloped patch covering dorsum, covered with metallic scales. A patch of bright white setae sits above the anal tubercle.

**Female** (paratype). ***Carapace*** length 1.36; ***abdomen*** length 1.89. Much darker than the male in body and appendages (Figs 130, 131). ***Carapace*** dark brown, black in ocular area, sparsely covered with paler scales. ***Clypeus*** and ***chelicerae*** brown, more or less glabrous. ***Chelicerae*** brown, more or less. ***Palps*** and ***legs*** honey coloured but with strongly contrasting black markings: annulae at joints, black stripes or patches on front and back faces of femora, and black stripe on front face of first and second tibiae. Abdomen black but with reflective metallic scales. ***Epigyne*** (Figs 110, 111) with distinctive dark inverted “V” in which are the narrow openings into the copulatory ducts, though lateral pockets may lead the embolus to the openings.

####### Additional material.

22 males and 6 females from: Ecuador: Napo: Tarapoa. 23 June – 1 July 1988 W. Maddison WPM#88-002 (1 male); Ecuador: Napo: bridge over Rio Cuyabeno on road to Tipishca. 25–30 June 1988 W. Maddison WPM#88-004 (1 male 1 female); Ecuador: Napo: bridge over Rio Cuyabeno on road to Tipishca. 29–30 July 1988 W. Maddison WPM#88-018 (4 males 2 females); Ecuador: Napo: Reserva Faunistica de Cuyabeno, Laguna Grande, Sendero La Hormiga. 2–5 August 1988 W. Maddison WPM#88-023 (2 males); Ecuador: Napo: Reserva Faunistica de Cuyabeno, Laguna Grande, PUCE field station. 1–7 August 1988 W. Maddison WPM#88-025 (1 male); Ecuador: Napo: bridge over Rio Cuyabeno on road from Lago Agrio to Tipishca. 8–9 August 1988 W. Maddison WPM#88-027 (1 male); Ecuador: Sucumbios: Reserva Faunistica Cuyabeno, Laguna Grande, PUCE field station. 0.002, -76.172. 21–29 July 1989 W. Maddison WPM#89-032 (1 male); Ecuador: Sucumbios: bridge over Rio Cuyabeno on road between Tarapoa and Tipishca, 0.025, -77.308. 29 July 1989 W. Maddison WPM#89-036 (1 male); Ecuador: Sucumbios: Reserva Faunistica Cuyabeno, Nuevo Mundo cabins along Rio Cuyabeno at jcn with Lago Agrio-Tipishca HWY 19–29 April 1994 W. Maddison WPM#94-021 (3 males); Ecuador: Sucumbios: Reserva Faunistica Cuyabeno, Nuevo Mundo cabins, jcn Rio Cuyabeno & Lago Agrio-Tipishca HWY tree trunks 19–29 April 1994 W. Maddison WPM#94-023 (1 male); Ecuador: Morona Santiago: km 3 from Limón towards Gualaceo. 2.9663, -78.4209; 1250 m el. 12 July 2004 Maddison, Agnarsson, Iturralde, Salazar. WPM#04-030 (1 male 2 females); Ecuador: Morona Santiago: km 4 from Limón towards Gualaceo. 2.9808, -78.4414; 1380 m el. 12 July 2004 Maddison, Agnarsson, Iturralde, Salazar. WPM#04-031 (2 males); Ecuador: Orellana: Yasuní Res.Stn.area, Station area 0.675, -76.397 210–280 m elev. 26 July – 13 Aug 2011 Maddison/Piascik/Vega WPM#11-015 (2 males); Ecuador: Orellana: Yasuní Res.Stn.area, Station area 0.674, -76.397 210–280 m elev. Clearings, forest edge 8–9.Aug.2011 Maddison/ Piascik/Vega. WPM#11-104 (1 male); Ecuador: Orellana: Río Bigal Reserve, boundary along road. 0.541, -77.424. 970 m elev. 5 November 2010. M Vega, D & W Maddison, M Reyes. WPM#10-048 (1 female). (Note: the province Sucumbios was established after 1988; the 1988 localities listed as Napo Province would now all be in Sucumbios.).

##### Genus *Sittisax* Prószyński, 2017, restored (removed from synonymy with *Sitticus*)

*Sittisax* Prószyński, 2017 (type species *Euophrys saxicola* C.L. Koch, 1846)

Breitling’s (2019) synonymy of *Sittisax* with *Sitticus* is here rejected based on our phylogenetic results, which strongly support it as the sister group of *Tomis*. According to the phylogeny, this lineage of two species arrived from the New World to Eurasia independently from *Attulus*, and retains a few features more similar to the other members of the *Jollas*-*Tomis* clade: the RTA is offset basally, and the epigynal openings are placed anteriorly and medially.

###### Sittisax ranieri

Taxon classificationAnimaliaAraneaeSalticidae

(Peckham & Peckham, 1909)

46E57CD3-239F-5A1C-B655-A2C9FDAB8F2B

Figures 99–103

Attus lineolatus Grube, 1861 (junior homonym)
Sittacus ranieri Peckham & Peckham, 1909

####### Remarks.

The Holarctic *Sittisax ranieri* is a widespread boreal species, which in North America follows the high elevations of the Western Cordillera to the south, living on rocks and litter. It is dark in colour, large-bodied, and with distinctive genitalia.

####### Material examined.

Canada: Northwest Territories: Tuktoyaktuk (1 male); Nunavut: Baffin Island (1 female); British Columbia: Downton Creek (2 males 2 females), 49.026, -114.061 (1 male), 59.8, -127.5 (1 male), Pink Mountain (1 male); Yukon: km 72 Dempster Highway (2 males, 2 females); km 75.6 Dempster Highway (1 female); Saskatchewan: 55.27, -105.19 (2 males), Ontario: Old Woman Bay (1 female); New Brunswick: 65.336, -69.4 (6 males, 3 females); U.S.A.: Washington: Spray Park (1 males, 2 females); Oregon: 45.261, -117.178 (1 female); Colorado: 39.803, -105.782 (1 male).

##### Genus *Tomis* F.O. Pickard-Cambridge, 1901, restored (removed from synonymy with *Sitticus*)

Figures 5, 6, 106, 107, 120–128

*Tomis* F.O. Pickard-Cambridge, 1901 (type species *Tomis palpalis* F.O. Pickard-Cambridge, 1901)

*Pseudattulus* Caporiacco, 1947 (type species *Pseudattulus kratochvili* Caporiacco, 1947), syn. nov.

**Figures 120–128.**
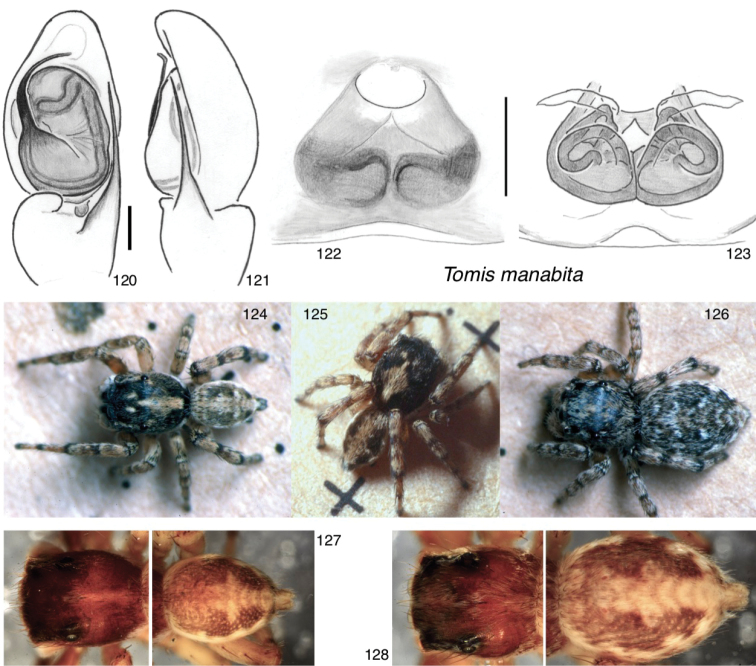
*Tomis manabita*, sp. nov. **120, 121** Left palp of holotype **120** ventral view **121** retrolateral view **122** ventral view of epigyne of paratype **123** dorsal view of same, cleared **124–128** specimens from type locality **124** male **125** male **126** female **127** male holotype **128** female paratype.

A Neotropical group whose male spermophore (with some possible exceptions) has a “shortcut loop”. That is, the large loop of the spermophore that normally occupies much of the visible face of the tegulum, and which points basally in many sitticines (e.g., Fig. 89), is incomplete, instead diving into the subtegulum, and thus not returning terminally to complete the loop on the surface of the tegulum (e.g., Fig. 120; Galiano 1991a: fig. 13).

The phylogeny strongly places *T. palpalis*, *T. manabita*, and *Sittisax ranieri* together. Although the phylogeny gives us the freedom to synonymize *Sittisax* into *Tomis*, this deep clade will eventually deserve at least two genera, and so we tentatively retain the boundary between the Neotropical *Tomis* and the Holarctic *Sittisax*, based on the apparent difference in spermophore loops. The other species are included in *Tomis* because of their apparent relationship with *T. palpalis* and *T. manabita*. The *palpalis* group (*T. palpalis*, *T. canus*, *T. mazorcanus*, *T. phaleratus*, *T. vanvolsemorum*, and *T. uber*) is delimited by a flattened cymbium (Galiano, 1991a) and well-separated epigynal openings. The remaining species all are known from coastal areas of the Caribbean or South America, and at least some live on beaches. They might not form a monophyletic group, as some show a long thin RTA, others not. *T. pavidus* and *T. mona* appear especially close to *T. manabita* by similarities in the palps. The others can be tentatively included in *Tomis* because they share with *T. palpalis* and *T. manabita* the shortcut spermophore loop.

The placement of *Sitticus welchi* Gertsch & Mulaik, 1936 in *Tomis* is tentative. The holotype female (AMNH, examined) lacks most of its legs and setae, but is nonetheless identifiable as a sitticine through the absence of a retromarginal cheliceral teeth and a very long leg that appears to be (it is disarticulated) of the fourth pair. The single anteriorly-placed epigynal opening (Figs 106, 107) indicates it belongs in the *Jollas*-*Tomis* clade. What suggests placement in *Tomis* in particular is the deep incision from the epigastric furrow toward the epigynal opening (Fig. 106). Such an incision as seen also in *Tomis mona* (Bryant 1947: fig. 6), which itself is placed in *Tomis* by the close similarity between its palp and that of *T. manabita*.

We synonymize *Pseudattulus* (see Ruiz et al. 2007) based on its shortcut spermophore loop and flattened cymbium, which suggest close relationship to (or membership in) the *palpalis* group. We accept (and thus re-assert) Ruiz et al.’s (2007) synonymy of *Sitticus cabellensis* Prószyński, 1971 with *Pseudattulus kratochvili*. Prószyński (2017a) had rejected their synonymy, but we see no basis for this, as Ruiz et al. had explained it well. Although we suspect *Pseudattulus* will eventually be reinstated, keeping it separate now would most likely yield a non-monophyletic genus *Tomis*. For many species (e.g., those from the Galapagos) we have no basis for choosing whether to assign them to *Pseudattulus* or to *Tomis*, and so either or both genera, if separated, would likely be non-monophyletic. Uniting them solves this until we have better phylogenetic information.

###### Tomis manabita

Taxon classificationAnimaliaAraneaeSalticidae

W. Maddison sp. nov.

10AE5227-3E2F-52CF-A4AD-23408A5379DA

http://zoobank.org/4C656386-8B15-4B5C-BF3F-27805897C65F

Figures 120–128

####### Type material.

Male holotype, 10 male and 8 female paratypes from Ecuador: Manabí: Puerto Rico, Cabañas Alandaluz 5 May 1994 W. Maddison WPM#94-031. The holotype (specimen UBC-SEM AR00217) pertains to the Museum of Zoology, Pontificia Universidad Católica, Quito, Ecuador (QCAZ), but is currently held in the Spencer Entomological Collection at the Beaty Biodiversity Museum, University of British Columbia (UBC-SEM).

####### Etymology.

Based on the type locality; the form is the adjective in Spanish (masculine or feminine).

A species on the beaches of coastal Ecuador, resembling *Attulus* in habitus. It was used in the molecular phylogenetic study of Maddison and Hedin (2003) under the name “*Sitticus* sp.” (voucher S220) from Manabí, Ecuador.

####### Diagnosis.

Palp closely resembles that of *Tomis pavidus*, from which it differs in the smaller tibia and longer RTA.

####### Description.

**Male** (holotype). Carapace length 1.58; abdomen length 1.51. ***Carapace*** (Figs 124, 127) medium brown with recumbent brown setae except for thin medial longitudinal band of white setae on thorax, two spots of pale setae in ocular area, and a marginal band of white setae that is broad on the thorax but narrows to the anterior, disappearing before the clypeus is reached. ***Clypeus*** brown, with a few brownish setae. ***Chelicerae*** vertical, with a few pale setae near the clypeus. Retromarginal cheliceral teeth lacking. ***Palp*** clothed with white setae dorsally, but prolaterally with darker integument and setae from tip of femur to cymbium; cymbium mostly dark brown. ***Embolus*** (Fig. 120) arises broadly, more centrally beneath bulb (and less peripherally) than is typical, then narrows abruptly at ca. 9 o’clock. RTA extremely long and thin, parallel to axis of the palp. ***Legs*** honey-coloured, with notably darker annulae at the tarsus-metatarsus joints, and black stripe on prolateral-ventral face of first patella and tibia. ***Abdomen*** (Figs 124, 127) brown with two lateral and one median longitudinal bands of paler setae, the medial band less distinct, wavy, and accompanied by a lateral extension that forms a cross.

**Female** (paratype # UBC-SEM AR00218). ***Carapace*** length 1.76; ***abdomen*** length 2.22. ***Carapace*** (Figs 126, 128) brown, covered unevenly with recumbent cream-coloured setae. ***Clypeus*** with white setae, longest at midline where they overhang the chelicerae. Chelicerae brown, with a few setae near clypeus. ***Palps*** and ***legs*** honey coloured, with weak annulae. ***Abdomen*** brown, marked as in male except bands are less distinct (Figs 126, 128). ***Epigynum*** with an anterior atrium from which short copulatory ducts lead diagonally to the spermathecae (Figs 122, 123).

####### Additional material.

15 males and 7 females from Ecuador: Manabí: Machalilla National Park, Salaite, between HWY and coast 6 May 1994 W. Maddison WPM#94-032 (4 males, 2 females); Ecuador: Manabí: Machalilla National Park, Salaite, 1 km inland along trail from HWY. 6 May 1994 W. Maddison WPM#94-033 (3 males); Ecuador: Manabí: Machalilla National Park, trail between Agua Blanca & San Sebastien 50–400 m dry forest 7 May 1994 W. Maddison WPM#94-034 (1 male); Ecuador: Manabí: Crucita. 30 August 1988 W. Maddison WPM#88-040 (2 males 4 females); Ecuador: Del Oro: Jambelí 13 August 1989 W. Maddison WPM#89-040 (3 males); Ecuador: Manabí: Puerto Lopez. 1.5497, -80.8104; 5 m el. 1–5 August 2004 W. Maddison. WPM#04-067 (2 males 1 female).

#### Species misplaced as sitticines

The following species are not sitticines, indicated by the presence of retromarginal cheliceral teeth (lacking in the Sitticini, a synapomorphy) or characteristic euophryine genitalia.

The following three are members of the Euophryini. They are left within sitticine genera because it is unclear to which genus they should be transferred.

*Jollas armatus* (Bryant, 1943)

*Jollas crassus* (Bryant, 1943)

*Jollas minutus* (Petrunkevitch, 1930)

The following two species described in *Sitticus* are also euophryines (see Prószyński 2017a). They are tentatively placed in a likely genus, *Chinophrys*:

*Chinophrys taiwanensis* (Peng & Li, 2002), comb. nov.

*Chinophrys wuae* Peng, (Tso & Li, 2002), comb. nov.

The following species can be moved out of *Sitticus* to their appropriate genera. The type specimens of both, in the MCZ, have been examined.

*Heliophanus designatus* (Peckham & Peckham, 1903), comb. nov. – bears the stridulatory apparatus characteristic of chrysillines (Maddison 1987), as well as the body form, markings and epigynum typical of *Heliophanus*.

*Mexigonus peninsulanus* (Banks, 1898), comb. nov. – appears as a typical *Mexigonus* with euophryine genitalia.

## Chromosome diversity and evolution

### Chromosome observations

Table 2 summarizes the chromosome complements of the 18 sitticines studied along with those reported in the literature (Hackman 1948, Kumbiçak et al. 2014). Except in *Attinella concolor*, all autosomes are acrocentric. Eight species have the usual chromosome complement for male salticids, 13 pairs of acrocentric autosomes and X_1_X_2_0 sex chromosomes. Three taxa (*A. burjaticus*, *A. floricola*, *A. finschi*) have the standard XX0 sex chromosomes but an extra pair of autosomes to make 28a+XaXa0. Of the remaining species, six have neo-Y systems of varied forms, while the seventh, *Attinella concolor*, has apparently completed a series of Roberstonian fusions to generate all metacentrics and halve the chromosome number to male 14m + Xm0. The following account of our observations, species by species, gives the basis for our interpretation of chromosome complements.

**Table 2.** Chromosome complements observed for males of 17–18 species of sitticines. The autosomal counts represent diploid complement, and thus 26a means 13 pairs of acrocentric autosomes. In the chromosome counts, a = acrocentric (one-armed), m = metacentric (two-armed). Exx. is the number of specimens; nuc. is the number of nuclei showing the full chromosome complement; +nuc sex is the number of additional nuclei showing the sex chromosomes (though not clearly the autosomes). Uncertainties about scoring, in particular about *Attinella dorsata*, *Attulus burjaticus* and the specimen labelled “*Attulus rupicola/floricola*” are explained under Chromosome observations.

**Species**	**Autosomes**	♂ **Sex chrom.**	**Y present**	**Locality**	**exx**	**nuc**	+**nuc sex**
***Jollas*-*Tomis* clade**							
*Attinella concolor*	14m	Xm0	no	U.S.A.: Gainesville, 29.63, -82.37	1	6	2
*A. dorsata*	26a?	XaXa0?	?	U.S.A.: Dillon Cr., 41.57, -123.54	3	11	
*Jollas cupreus*	26a	XaXa0	no	Ecuador: Tarapoa, -0.12, -76.34	1	2	1
*Sittisax ranieri*	24a	XmXaYm	yes	Canada: Leguil Creek, 59.8, -127.5	2	10	1
				Canada: Inuvik, 68.31, -133.49	1		1
				Canada: Wawa, 47.79, -84.90	2	8	
				U.S.A.: Mt. Monadnock, 42.87, -72.11	1	3	
*Sittisax saxicola*	24a	XaXaXaYm or XmYaYaYa	yes	Switzerland: Flims, 46.9, 9.2	3	14	10
*Tomis manabita*	26a	XaXa0	no	Ecuador: Crucita, -0.9, -80.5	1	3	2
*** Attulus ***							
Attulus (Attulus) ammophilus	26a	XaXa0	no	Canada: Toronto, 43.65, -79.32	3	9	1
				Russia: Uvs Nuur, 50.6690, 92.9844	2	11	6
A. (A.) burjaticus	? (28a?)	XaXa0	no	Russia: Uvs Nuur, 50.677, 92.99	1	1	7
A. (A.) caricis	26a	XaXa0	no	38.6, 34.8 (Kumbiçak et al. 2014)	–		
A. (A.) cutleri	26a	XaXaYa	yes	Canada: Inuvik, 68.35, -133.70	1	3	4
A. (A.) floricola	28a	XaXa0	no	Canada: Barrie, 44.43, -79.65	1	7	7
				U.S.A.: Naselle, 46.43, -123.86	1	2	
*A. (A.) rupicola/floricola*	24a?	XaXaXaYm?	yes	Switzerland: Flims, 46.9, 9.2	1	3	5
A. (A.) inexpectus	26a	XaXa0	no	Russia: Uvs Nuur, 50.6690, 92.9844	2	13	5
A. (A.) striatus	24a	XaXaXaYm	yes	U.S.A.: Ponemah, 42.82, -71.58	1	5	6
Attulus (Sitticus) fasciger	26a	XaXa0		Canada: Burlington, 43.351, -79.759	3	16	
A. (S.) finschi	28a	XaXa0	no	Canada: Nipigon, 49.01, -88.16	1	4	
				Canada: Sault Ste. Marie, 46.94, -84.55	1	1	
				Canada: Chinook L., 49.67, -114.60	1	8	3
A. (S.) pubescens	26a	XaXmYa	yes	U.S.A.: Cambridge, 42.38, -71.12	4	10	9
A. (S.) terebratus	26a	XaXa0	no	Russia: Karasuk, 53.730, 77.866	1	9	14
	26a	XaXa0		Hackman (1948)	–		

### Chromosomes of the *Jollas*-*Tomis* clade

**Figures 129–139.**
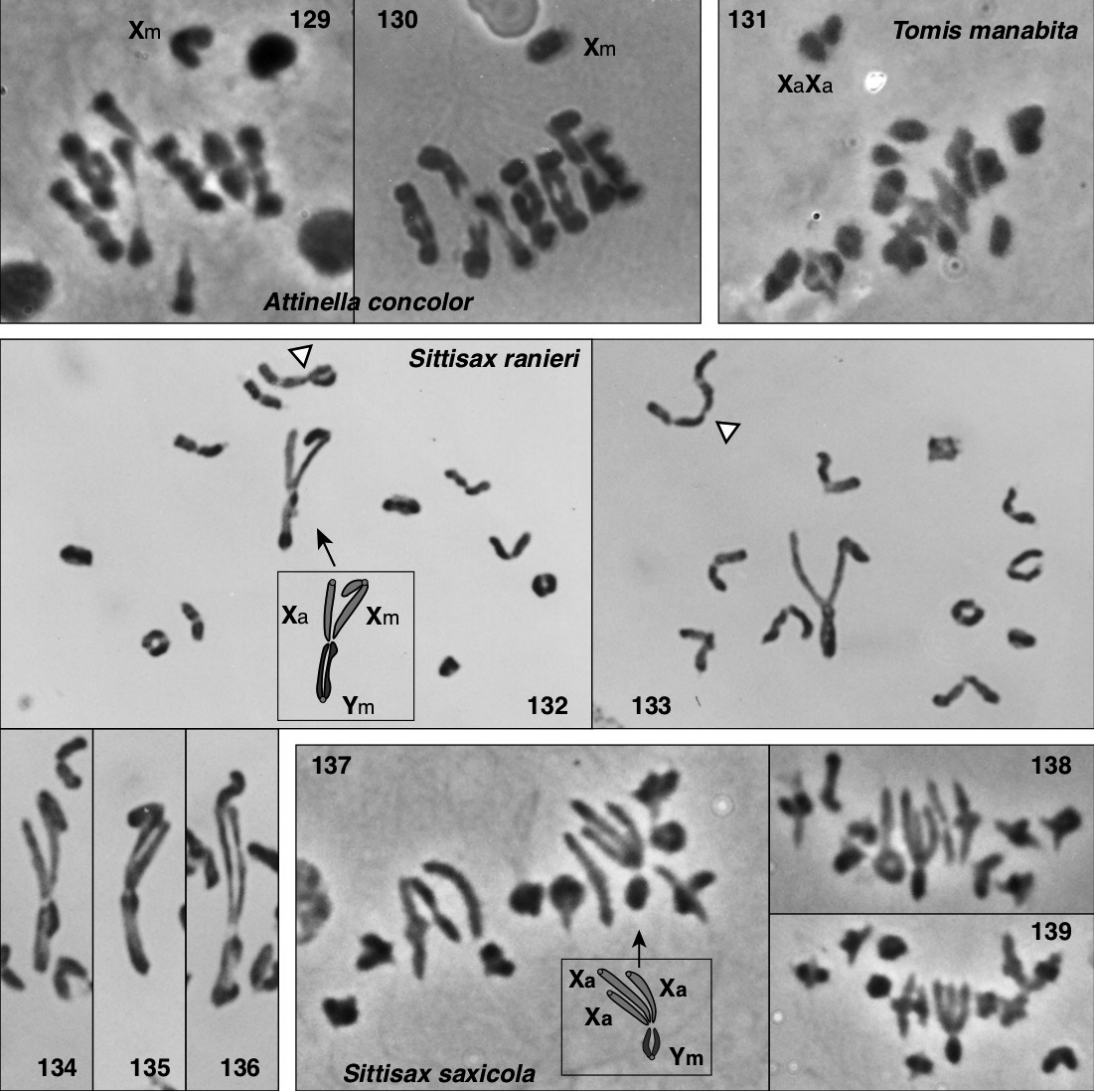
Chromosomes of first meiotic division in males of the *Jollas*-*Tomis* clade **129, 130***Attinella concolor*, with only seven pairs of autosomes, but each two-armed, 14m+Xm0, Florida (29.63N, 82.37W) **131***Tomis manabita*, showing the two Xs off to one pole, and 13 acrocentric bivalents on the metaphase plate, Ecuador (0.9S, 80.5W) **132–136***Sittisax ranieri*, whose distinctive XmXaYm appears as a rabbit head with a droopy ear. White triangles show points where two bivalents are apparently linked together **134–136** details of XXY of *S. ranieri***137–139***Sittisax saxicola*, with sex chromsomes, interpreted tentatively as XaXaXaYm, appearing as a rabbit head with three ears, Switzlerland (46.9N, 9.2E).

*Attinella concolor*: 14m+Xm0 (Figs 129, 130): Nuclei of first meiotic metaphase show clearly seven pairs of metacentric autosomes and one metacentric X chromosome (Figs 129, 130). The metacentric autosomes appear strikingly different from the usual acrocentrics typical of spiders. Most of the bivalents are held together by just one arm at first metaphase, the other free.

*Attinella dorsata*: 26a+XaXa0 (uncertain). Scored as 26a+XaXa0 in notes from the 1980s, the slides are too faded and degraded for precise re-count, but re-examination shows they have at least 13 acrocentric bivalents, and what looks like XX0. Although we might have abandoned the score entirely, we include it here to show that it is at least similar to the typical salticid complement, and not at all what is seen in the close relative *Attinella concolor*.

*Jollas cupreus*: 26a+XaXa0. One first division nucleus appears clearly as 13 autosomal bivalents plus two acrocentric Xs, while two more show the typical Xs side by side.

*Sittisax ranieri*: 24a+XmXaYm (Figs 132–136). The distinctive chromosome complement is confirmed by many clear nuclei. The sex chromosomes (Figs 132–136) appear as a rabbit’s head (the Y) with two ears (the Xs), one of which is floppy (a metacentric with an unpaired arm). The two arms of the metacentric Y and one of each X meet together at a single point, a junction of four arms. That the “head” and “ears” segregate to opposite poles is confirmed by second metaphase counts (nine nuclei with 12 acro.+1 meta.; five nuclei with 13 acro.+ 1 meta.). That the “head” is a Y and the “ears” are Xs is indicated by counts of 26 acrocentrics and two metacentrics in mitotic metaphase of two young females from Wawa, Canada (47.79N, 84.90W) (2 complete, countable nuclei found in each female, scored in 1986; now faded). Unlike *Habronattus* (Maddison, 1982) and most other species of sitticines, no heteropycnosis or achiasmate meiotic pairing was noted in the sex chromosomes of *S. ranieri* which would have indicated ancestral X material. We thus have no account for how this structure evolved, and what parts of it represent ancestral X versus autosome material.

*Sittisax saxicola*: 24a+XaXaXaYm or 24a+XmYaYaYa (good quality, though ambiguous in interpretation; Figs 137–139). The sex chromosome system in *Sittisax saxicola* is at least superficially similar to that in *S. ranieri* except that the “rabbit” has three straight “ears”. The many metaphase I nuclei observed show 12 clear autosomal acrocentric bivalents plus the sex chromosomes, while two mitotic nuclei had clear counts of 28 chromosomes, one of which is notably longer than the others, possibly the metacentric. The acrocentric “ears” of the sex chromosomes are always oriented together toward one pole at metaphase I, the metacentric “head” to the other, indicating either a XXXY or XYYY sex chromosome system. Consistent with this are three observations of second division nuclei with 15 acrocentrics, and one observation with 12 acrocentrics and a metacentric. There is no clear evidence from heteropycnosis, and no female karyotype, to indicate whether the “ears” are the Xs or the Ys. We might invoke parsimony to suggest the metacentric is the Y and the ears the Xs, as in *S. ranieri*, but will resist this, and treat the sex chromosomes as ambiguous, either XXXY or XYYY.

All four sex chromosomes of *S. saxicola* come together in a quintuple junction. This and the quadruple junction of *S. ranieri* are unusual, possibly formed because mutual translocations or repeated sequences generate a knit pattern of pairing. White (1965) postulated that a similar triple terminal junction in a mantid is formed by chiasmata joining the three arms on triple pairing segments and subsequently terminalizing. There is evidence that different autosomes in *Sittisax* might also have common terminal segments. In all males of *S. ranieri*, autosomal bivalents with proximal chiasmata are often joined together into tetravalent and sometimes hexavalent chains, via the terminal ends of one chromosome of each bivalent (see white triangles in Figs 132, 133). The terminal ends of the autosomes appear to have small satellites.

*Tomis manabita*: 26a+XaXa0 (Fig. 131). Although there are only a few nuclei, they show 13 autosomal bivalents plus two acrocentric Xs. In three nuclei, the two acrocentric Xs are side by side and off to one pole.

### Chromosomes of *Attulus*

**Figures 140–142.**
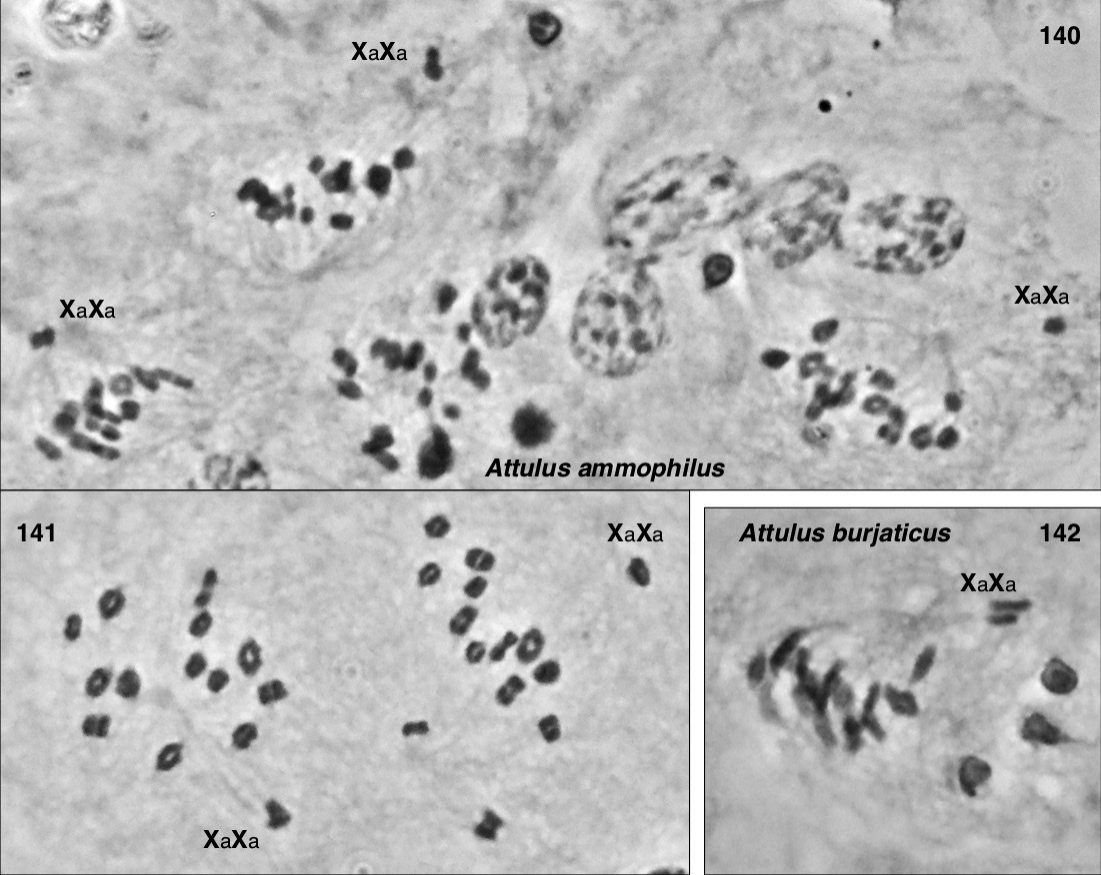
Chromosomes of first meiotic division of Attulus subgenus Attulus**140, 141***Attulus ammophilus*, Tuva (50.6690N, 92.9844E): **140** four nuclei, three showing the two X chromosomes toward one pole **141** two nuclei showing two Xs and thirteen pairs of acrocentric autosomes **142***Attulus burjaticus*, showing the two X chromosomes toward one pole, Tuva (50.677N, 92.99E). The three large spots to the lower right are spermatids.

Attulus (Attulus) ammophilus: 26a+XaXa0 (Figs 140, 141). Many clear nuclei show the classical 13 acrocentric bivalents and two acrocentric X’s off toward one pole.

Attulus (Attulus) burjaticus: ?+XaXa0 (autosome count uncertain; Fig. 142). One clear and isolated meiotic nucleus in metaphase I shows 15 figures, one of which is presumably be the XX, suggesting that it may have 28a+XX0. Six nuclei show a typical pair of XaXa toward one pole. The interpretation of XX0 seems reasonably secure, but the autosome count is not.

Attulus (Attulus) cutleri: 26a+XaXaYa (Figs 152, 153). There are a few clear nuclei, and several more in which the sex chromosomes are clear (but the autosome counts are not). Interpretation of the sex chromosomes seems fairly clear. They are interpreted to be XXY because two elements are seen side by side and slightly decondensed (the Xs). The third chromosome is small, paired terminally with the more condensed end of the larger X, and thus interpreted as a Y. There is no hint of a centromere in the larger X, and so all appear to be acrocentrics.

**Figures 143–153.**
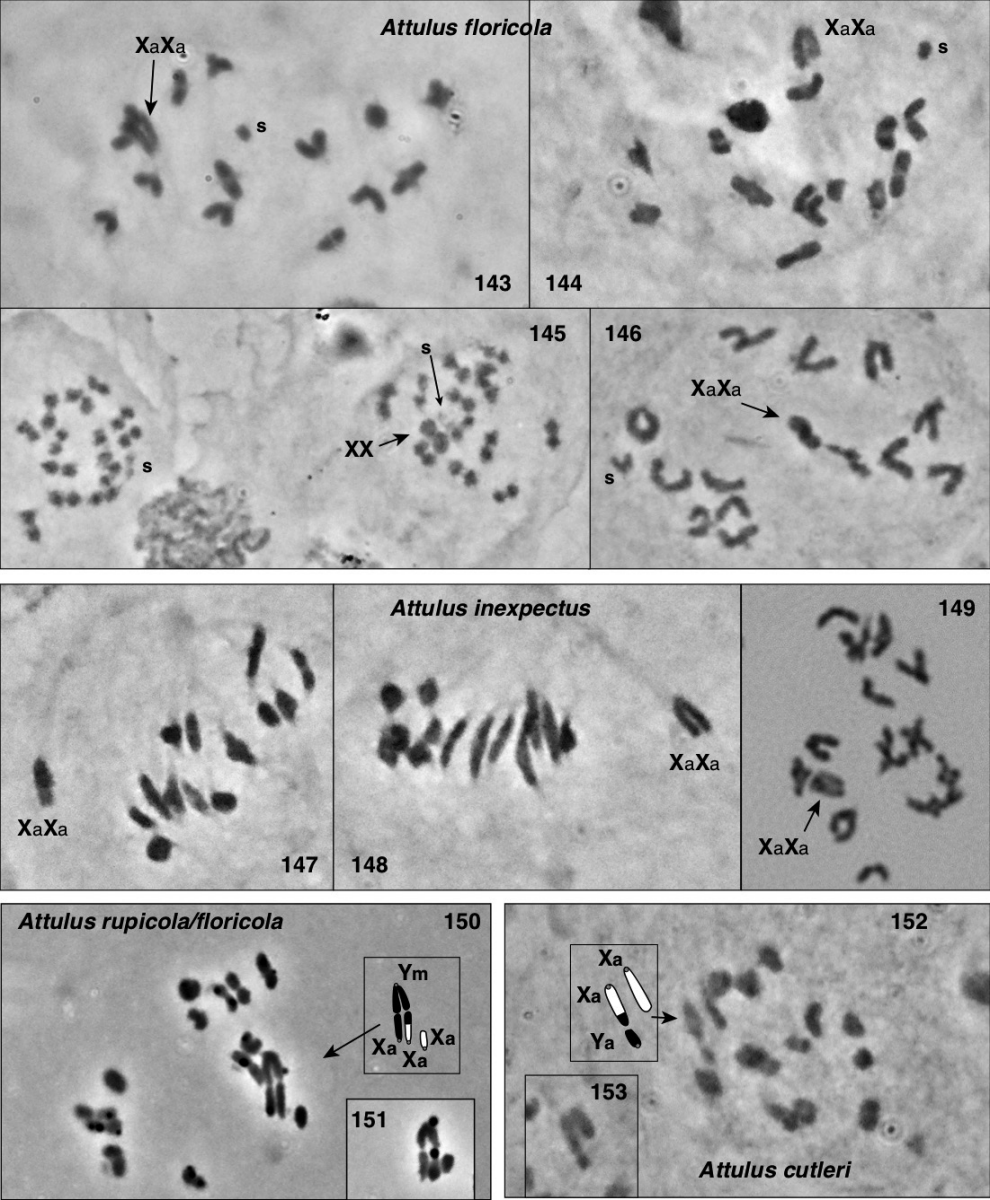
Chromosomes of meiosis of Attulus subgenus Attulus, continued **143–146***Attulus floricola*, with an extra small bivalent (**s**) to make 28a+XaXa0, Ontario (44.43, -79.65): **143, 144** first metaphase **145** second division, showing one nucleus with 14 acrocentrics, the other with 14 acrocentrics and the two condensed Xs **147–149***Attulus inexpectus*, showing 13 acrocentric bivalents and the sex chromosomes (26a+XaXa0), Tuva (50.6690, 92.9844) **150, 151***Attulus* sp. (ambiguously identified, either *A. rupicola* or *floricola*), tentatively intepreted as having 24a+XaXaXaYm, Switzerland (46.9, 9.2): **151** same, sex chromosomes from another nucleus **152***Attulus cutleri*, with 26a+XaXaYa, Canada (68.35, -133.70) **153** same, sex chromosomes from another nucleus

Attulus (Attulus) floricola: 28a+XaXa0, with one autosome much smaller (Figs 143–146). In addition to the clear division I nuclei showing the classic pair of X’s lying side by side, counts of second division nuclei show either 14 acrocentric chromosomes (six clear nuclei) or 16 chromosomes (five clear nuclei). All of the second division nuclei show one chromosome much smaller than the others. Those with 16 chromosomes show two of the chromosomes appearing larger and distinct in appearance, consistent with their being the Xs, pointing to an XaXa0 sex chromosome system.

*Attulus (Attulus) rupicola/floricola* (Switzerland): 24a+XaXaXaYm (uncertain in details, though the presence of at least one Y is secure; Figs 150, 151). The presence of a Y chromosome is well supported, but the details of the sex chromosome system are uncertain. No single nucleus shows both the chromosome count and the sex chromosome system convincingly. The total number of chromosomes (27 acrocentrics and one metacentric) can be seen in two mitotic nuclei, and in a few first division meioses. Although at least 20 nuclei show the V-shaped trivalent of metacentric (point of the “V”) and two acrocentrics (distal arms of the “V”), interpreted as the Y and two Xs, only three show the fourth member, an acrocentric, lying near one of the Xs. This achiasmate association leads us to intepret the system as XaXaXaYm rather than XmYaYaYa, but the evidence is weak, as there are no female counts, heteropycnosis is not obvious, and most often the fourth member is lying distant from the trivalent, usually not obviously directed to the same pole as the two acrocentrics, though not apparently oriented against it either.

Attulus (Attulus) inexpectus: 26a+XaXa0 (Figs 147–149). Several very clear first division nuclei show 13 acrocentric bivalents and the two acrocentric Xs, heteropycnotic and lying side by side, off of the metaphase plate. Three second division counts are consistent with an XX0 sex chromosome system (two counts of 13 acrocentrics; one count of 15).

Attulus (Attulus) striatus: 24a+XaXaXaYm. The slides are too faded to score now even under phase contrast, and so for this we rely entirely on notes from 1985. Those notes give good evidence to consider the interpretation secure. The slides were then clear enough to score chiasma localization in the acrocentric autosomes (in 14 nuclei with at least ten autosomes scorable, the numbers of proximal vs. interstitial vs. terminal chiasmata were 76:12:50 respectively). Five of these nuclei showed a clear count of 14 acrocentric autosomes. The sex chomosomes were clear in several nuclei, consisting of a “V” shaped trivalent with a metacentric at the point of the “V”, to each arm of which was paired an acrocentric. One of those acrocentrics was decondensed (heteropycnotic) in its centromeric half, and lying alongside it achiasmately was a decondensed acrocentric, thus in total making a figure of four. The achiasmate pairing and heteropycnosis suggest those acrocentrics have ancestral X material, as in the XXXY *Habronattus* (Maddison 1982, Maddison & Leduc-Robert 2013), which this resembles strongly. Three pairs of second division nuclei showed one member with 15 acrocentrics, the other with 12 acrocentrics and a metacentric. Together this points to an XaXaXaYm sex chromosome system.

**Figures 154–164.**
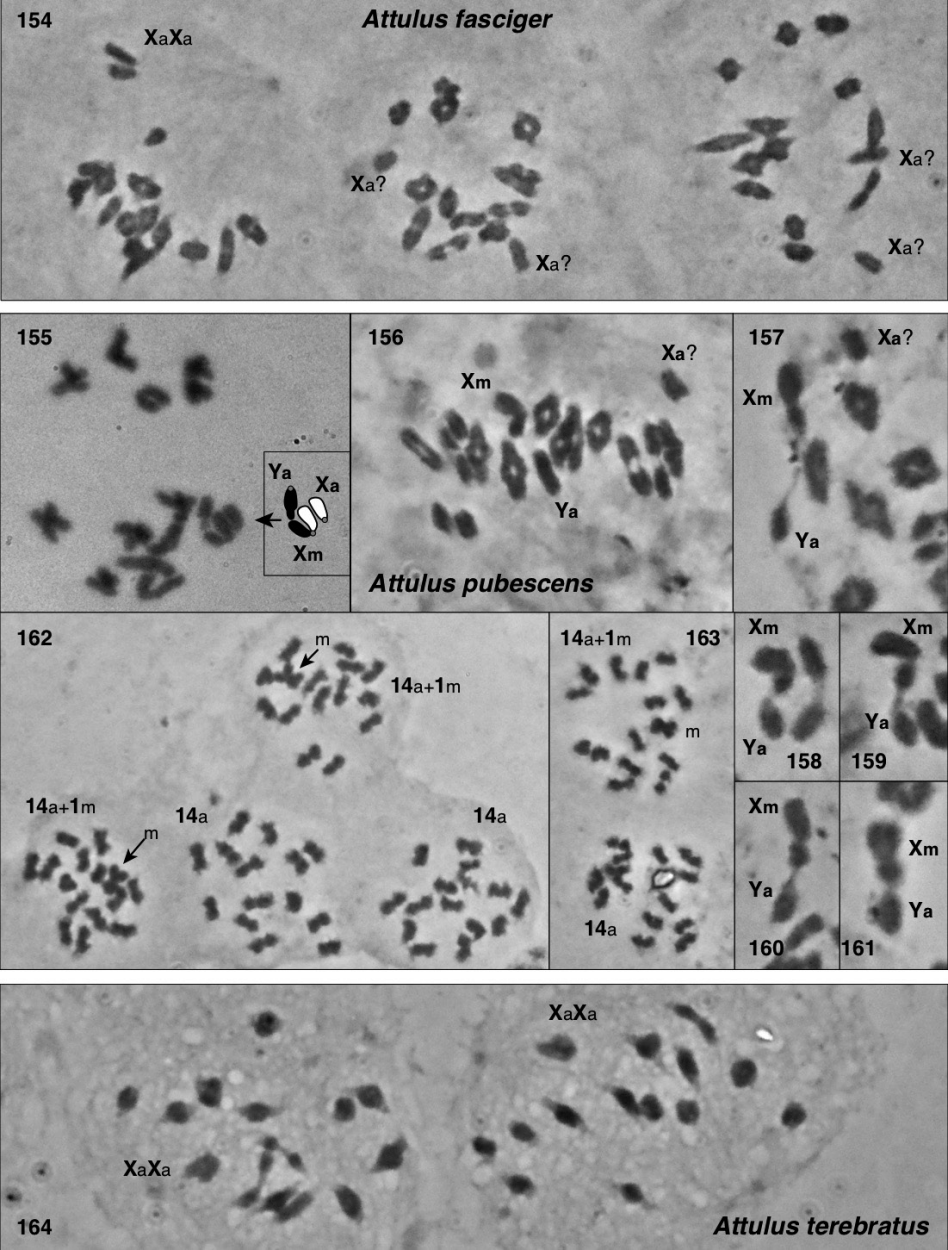
Chromosomes of meiosis of Attulus subgenus Sitticus**154***Attulus fasciger*, three nuclei, one showing the two Xs together and toward a pole, Canada (43.351N, 79.759W) **155–163***Attulus pubescens*, with XaXmYa sex chromosomes, Massachusetts (42.38N, 71.12W) **157–161** XmYa sex chromosomes from other nuclei; the second X is often not paired with them **162, 163** Second division nuclei, all having 14 acrocentrics, and some having in addition a metacentric (m) **164***Attulus terebratus*, two nuclei (26a+XaXa0), Novosibirsk Oblast (53.730N, 77.866E).

Attulus (Sitticus) fasciger: 26a+XaXa0 (Fig. 154). Many clear nuclei show the classical 13 acrocentric bivalents and two acrocentric X’s (heterpycnotic, side by side or apart) off toward one pole. A few division-2 nuclei are consistent with this (three nuclei with 13 similar acrocentrics; one nucleus with 13 similar and two more condensed acrocentrics).

Attulus (Sitticus) finschi: 28a+XaXa0, with one autosome much smaller. This score relies primarily on old notes, which indicate 28 acrocentric autosomes, one much smaller than the others, and two acrocentric Xs. From the Chinook Lake specimen we have been able to re-score eight nuclei in first division with 15 figures, all appearing as acrocentrics, and one much smaller than the others. The quality of those nuclei is now too poor to distinguish the Xs. However, three other metaphase nuclei in which the autosomes are not countable show clearly the two acrocentric Xs heteropycnotic and lying side by side and toward one pole.

Attulus (Sitticus) pubescens: 26a+XaXmYa (Figs 155–163). Many nuclei indicate 26 acrocentric autosomes, but relatively few show the sex chromosomes clearly, either because they are folded over themselves, or the X_2_ is not clearly associated with the others. However, many first division nuclei show a peculiar figure with a metacentric (X_1_) whose shorter arm is paired terminally with an acrocentric (Y). The longer arm of the X_1_ is heteropycnotic, and is occasionally seen with the X_2_ lying achiasmately beside it. This behaviour suggests that the metacentric and loose acrocentric are X’s, and this is supported by two cases of paired second division nuclei: in each, one of the pair shows 14 all-acrocentric chromosomes, while its partner shows more than 14 chromosomes, two of which are heteropycnotic. All though the latter were not fully countable, in total 24 second division nuclei were countable, 12 with 14 acrocentrics, and 12 with 14 acrocentrics plus a metacentric. Together these point to one metacentric and one acrocentric X going to one pole, in addition to 13 acrocentric autosomes, and one acrocentric Y to the other.

Attulus (Sitticus) terebratus: 26a+XaXa0 (Fig. 164). Several well-spread first metaphase show the two acrocentric Xs side by side and off to one pole, accompanied by 13 pairs of acrocentric autosomes. Two second division nuclei show 15 acrocentrics, two of which are especially condensed (thus, the Xs), while one shows 13 normal acrocentrics.

### Chromosome evolution

**Figure 165.**
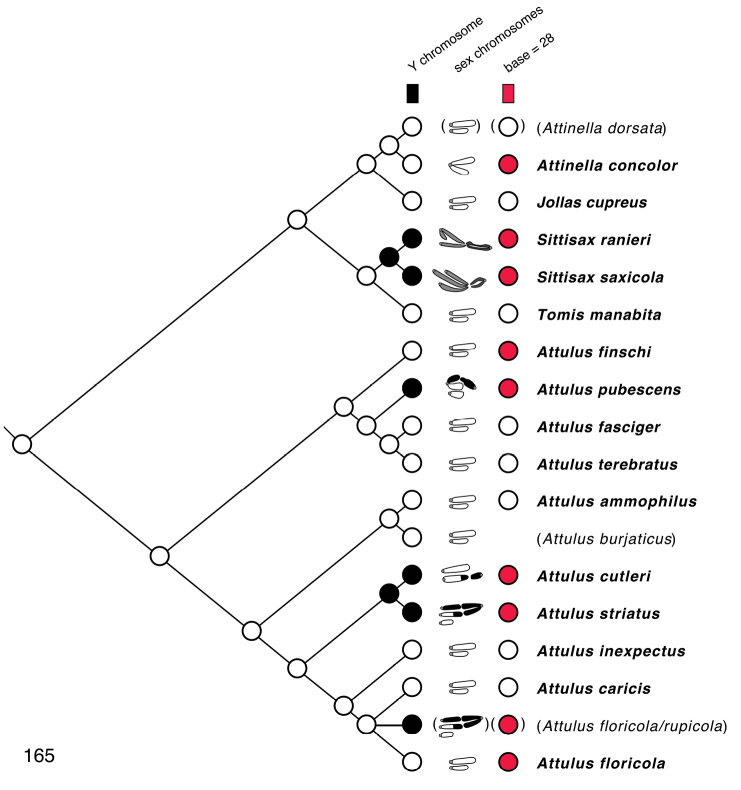
Chromosome evolution in sitticines. Ancestral nodes show the most parsimonious reconstruction of the evolution of Y via X-autosome fusions (black) from the X_1_X_2_0 sex chromosome system (white). Phylogeny from Figure 48 with species added as follows: *Attinella concolor* is very similar in body and genitalia to *A. dorsata*; likewise *Sittisax saxicola* to *S. ranieri*; *Attulus caricis* position based on COI results (Fig. 96). The similar pair *A. cutleri* and *A. striatus* were placed as sisters to the *floricola* group based on their inclusion in the *floricola* group by Logunov and Kronestedt (1997) and in *Sittiflor* by Prószyński (2017a). Base chromosome number is directly the number of autosomes if the species has XX0 sex chromosomes, but is interpreted as the number of autosomes +2 if the species has XXY sex chromosomes (apparently derived by a single fusion that would have consumed an autosomal pair), or + 4 if XXXY (apparently derived by two fusions that would have consumed two pairs). Uncertain scoring is shown by parentheses (see Table 2).

While salticids are fairly conservative in basic chromosome complement, with most species showing 26 acrocentric autosomes and X_1_X_2_0 sex chromosomes (Maddison 1982; Araujo et al. 2016, Araujo et al. 2019), sitticines are striking for their diversity. The distribution of chromosome complements on the reconstructed phylogeny (Fig. 165) suggests that neo-Y chromosomes arose four separate times; the alternative, assuming a Y was ancestral, is much less parsimonious, requiring seven losses to XX0. Outgroups also favour XX0 as ancestral in sitticines: it is very much the most common sex chromosome system in salticids, and the alternatives are phylogenetically scattered, with no known Y chromosomes in other amycoids (Maddison 1982; Araujo et al. 2016, Araujo et al. 2019). Four X-autosome fusions among 18 species represents a phylogenetic density approximately as high as in *Habronattus* (Maddison and Leduc-Robert 2013), but the resulting forms of sex chromosomes are more varied in *Sitticus*.

The ancestral autosome number in sitticines is unclear. Among the species with XX0, some have 26 autosomes, others have 28. Assessing a comparable autosome number with neo-Y species requires interpretation, as the neo-Y system itself binds one or more autosomal pairs with the X chromosomes, as indicated in part by distinctive condensation patterns. If (as in *Habronattus*, Maddison and Leduc-Robert 2013) we interpret the XXY systems as having one pair of autosomes bound into the sex chromosomes, and XXXY as having two pairs, then (for example) the 26a+XaXaYa of *A. cutleri* is interpreted as having a base number of 28 (26 free and two bound). The rightmost (red and white) column of Fig. 165 shows these interpreted base numbers. The most parsimonious interpretation would then consider that red (28) is ancestral for the entire clade of *Attulus*, reverting back to the typical salticid number (26) multiple times. The ancestral node of the *Jollas*-*Tomis* clade, and the root of the Sitticini, could be 26 or 28 equally parsimoniously if the expected outgroup condition of 26 were not imposed.

An unanticipated but consistent correlation between base autosome number and the presence of neo-Y is seen in Fig. 165, regardless of how we interpret the ancestral state for base autosome number. The pattern is phylogenetically repeated: each of the four separate neo-Y origins occurs in a 28-autosome lineage, and for each the closest lineage with 26 has XX0. We have no suggestion as to why there might be such a correlation. This pattern is unlikely to be a tautological consequence of our counting rule that interprets XXY/XXXY systems as incorporating two/four autosomes. The counting rule is derived (partially) independently, from condensation patterns and meiotic orientation. Even lacking an independent argument within sitticines, we could import the counting rule from *Habronattus*, where such an interpretation is well supported by meiotic behaviour and chromosome counts (Maddison 1982, Maddison and Leduc-Robert 2013). We do not know how to explain a correlation between an extra pair of autosomes and the presence of neo-Y, but it is perhaps relevant that in all of the 28a+XaXa0 species, one of the chromosome pairs is especially small, half or less the size of the others.

If these small chromosomes are supernumerary (B) chromosomes, it is possible that there is considerably more variation within species than our small sample sizes can detect. Undetected intraspecific variation in autosomes or sex chromosomes would not negate our basic evolutionary conclusions. Were we to find species variable with respect to the presence of a neo-Y chromosome, for example, it would point to even more transitions between XX0 and XXY/XXXY.

Our uncertainty about chromosome complement in some species does not strongly affect our conclusions about homoplasy or correlations, though it could affect a detailed reconstruction of the evolution of autosome number, or of particular fusions involved in a neo-Y system. For instance, if we delete autosome number for *Attinella dorsata* and *Attulus burjaticus* (the two species with uncertain counts) from Fig. 165, the ancestral states reconstructed by parsimony become ambiguously 28 or 26. Although we are uncertain about the detailed intepretation of sex chromosomes in *A. rupicola/floricola* and *Sittisax saxicola*, we conclude that they do have Y chromosomes, and thus the reconstruction of Y chromosome evolution is not affected. The scope of uncertainty allows one possible contradiction to our assessments above: should we be incorrect about the autosome count of *A. rupicola/floricola*, this may be a species in which a Y chromosome arose in the context of only 26 autosomes. Otherwise, the ambiguities do not change the interpretation of a correlation between a base number of 28 autosomes and neo-Y.

Chromosome evolution of sitticines will not be well understood, however, until a larger sample of species and specimens is obtained, given the high diversity seen in our small sample. Our data hint to the possibility of rapid evolution provoked by special mechanisms.

## Supplementary Material

XML Treatment for Aillutticina

XML Treatment for Attulus (Attulus) ammophilus

XML Treatment for Attulus (Attulus) floricola

XML Treatment for Attulus (Attulus) sylvestris

XML Treatment for Attulus (Attulus) striatus

XML Treatment for Attulus (Attulus) cutleri

XML Treatment for Attulus (Sitticus) finschi

XML Treatment for Attulus (Sitticus) fasciger

XML Treatment for Attulus (Sitticus) pubescens

XML Treatment for Attinella concolor

XML Treatment for Attinella dorsata

XML Treatment for Jollas cupreus

XML Treatment for Sittisax ranieri

XML Treatment for Tomis manabita
